# Syntheses and cytotoxicity of phosphatidylcholines containing ibuprofen or naproxen moieties

**DOI:** 10.1038/s41598-018-36571-1

**Published:** 2019-01-18

**Authors:** Marek Kłobucki, Anna Urbaniak, Aleksandra Grudniewska, Bartłomiej Kocbach, Gabriela Maciejewska, Grzegorz Kiełbowicz, Maciej Ugorski, Czesław Wawrzeńczyk

**Affiliations:** 1 Department of Chemistry, Wrocław University of Environmental and Life Sciences, Norwida 25, 50-375 Wrocław, Poland; 2 Laboratory of Glycobiology, Ludwik Hirszfeld Institute of Immunology and Experimental Therapy, Polish Academy of Sciences, Weigla 12, 53-114 Wrocław, Poland; 3 Faculty of Chemistry, Wrocław University of Science and Technology, Wybrzeże Wyspiańskiego 27, 50-370 Wrocław, Poland; 4 Department of Biochemistry and Molecular Biology, Wrocław University of Environmental and Life Sciences, Norwida 31, 50-375 Wrocław, Poland

## Abstract

In this study, novel phosphatidylcholines containing ibuprofen or naproxen moieties were synthesized in good yields and high purities. Under the given synthesis conditions, the attached drug moieties racemized, which resulted in the formation of phospholipid diastereomers. The comperative studies of the cytotoxicity of ibuprofen, naproxen and their phosphatidylcholine derivatives against human promyelocytic leukemia HL-60, human colon carcinoma Caco-2, and porcine epithelial intestinal IPEC-J2 cells were carried out. The results of these studies indicated that phospholipids with NSAIDs at both *sn*-1 and *sn*-2 positions (15 and 16) were more toxic than ibuprofen or naproxen themselves, whereas 2-lysophosphatidylcholines (7 and 8) were less toxic against all tested cell lines. Phospholipids with NSAIDs at *sn*-1 and palmitic acid at *sn*-2 (9 and 10) were also less toxic against Caco-2 and normal cells (IPEC-J2).

## Introduction

**Figure 1:**
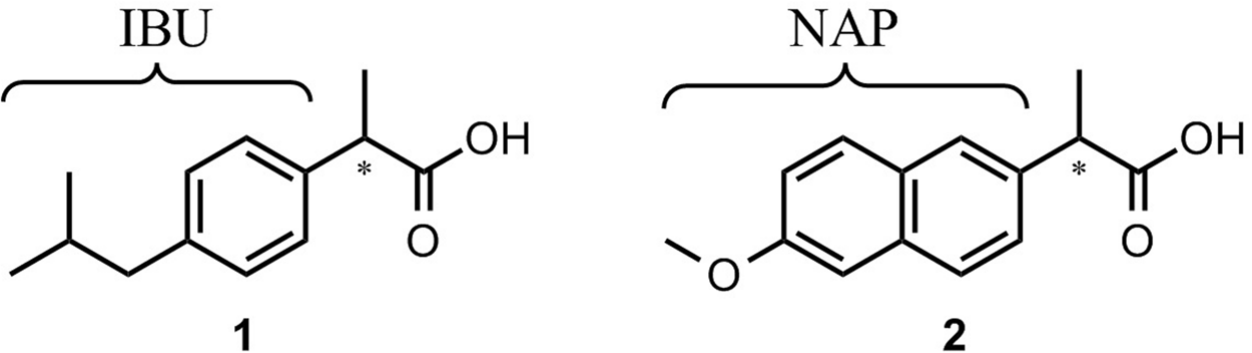
Structures of ibuprofen (**1**) and naproxen (**2**). IBU and NAP are shortcuts of ibuprofen and naproxen moieties used in the following schemes.

Nonsteroidal anti-inflammatory drugs (NSAIDs) exhibit anti-inflammatory, analgesic, and antipyretic effects^1,2^ and are currently some of the most widely used drugs in the world. NSAIDs are a large heterogeneous group of compounds and their classification is based mainly on their chemical structure. The most well-known group of NSAIDs are derivatives of 2-arylpropionic acid (profens) such as ibuprofen (**1**) and naproxen (**2**) (Fig. 1). The fact that these over-the-counter (OTC) drugs are readily available and relatively inexpensive is what makes them first-line drugs (FLD) for pain relief and inflammation treatment.

Only *S* isomers of profens consistently bind to proteins and inhibit prostaglandin synthesis^3,4^. *In-vivo* studies have shown that in some animal organisms, including humans, the inactive *R* enantiomer can be converted to a pharmacologically active *S* isomer by 2-arylpropionyl-CoA epimerase^4,5^. Since the synthesis of optically active drugs is costly, the production and sale of racemic mixtures has been approved. However, preparations containing drugs as (*S*)-isomers are also available, e.g., dexibuprofen and dexketoprofen^6,7^.

The mechanism of action of all NSAIDs is closely related to the inhibition of the enzyme cyclooxygenase-2 (COX-2). Unfortunately, most drugs, including ibuprofen (**1**) and naproxen (**2**), show lack of selectivity and inhibit the cyclooxygenase-1 (COX-1) enzyme, which is involved in maintaining the normal homeostasis of the body^8^. Therefore, long-term use of ibuprofen (**1**) or naproxen (**2**) has negative effects on the gastrointestinal tract (GI), leading to damage of the mucous membrane and consequent bleeding and perforation^8,9^.

Because of this GI toxicity, there is a clear need to create new generations of NSAIDs. In recent years, many NSAID derivatives have been found to exhibit similar activities but with less side effects than standard drugs. From these, noteworthy are the nitric oxide-releasing prodrugs of NSAIDs (NO-NSAIDs)^10,11^ and the derivatives of NSAIDs with phosphate moieties (phospho-NSAIDs)^12^. Moreover, it has been reported that phospho-NSAIDs are safer than normal drugs because of their reduced gastrointestinal toxicity^13^. In addition to their anti-inflammatory properties, it has been found that phospho-NSAIDs exhibit anticancer properties against colon and breast cancer^12,14–16^.

Among different approaches to improving drug quality, there has been a huge potential for drugs combined with phospholipids^17–19^. One reason for this is that they are non-toxic and biocompatible. Studies that were carried out on animal and human cells indicated that the mixture of NSAIDs and phosphatidylcholine exhibits stronger activity at identical concentrations and lower GI toxicity than NSAIDs alone^19–22^. Moreover, phospholipids with linked drugs mediate the transport of compounds and increase their bioavailability^23^. Our studies showed that compounds with anticancer activity linked covalently with phosphatidylcholine in the place of natural fatty acids lose their toxicity toward normal cells^24,25^.

In this paper, we report the synthesis of phosphatidylcholines containing ibuprofen (**1**) or naproxen (**2**) at *sn*-1 and/or *sn*-2 positions. Phosphatidylcholines with ibuprofen have previously been reported^26^ but herein we applied a different, shorter synthetic pathway. Additionally, the cytotoxicity of the newly synthesized phospholipid derivatives against human promyelocytic leukemia HL-60 cells, human colon carcinoma Caco-2 cells, and porcine epithelial intestinal IPEC-J2 cells was studied.

## Results and Discussion

### Chemistry

**Figure 2:**
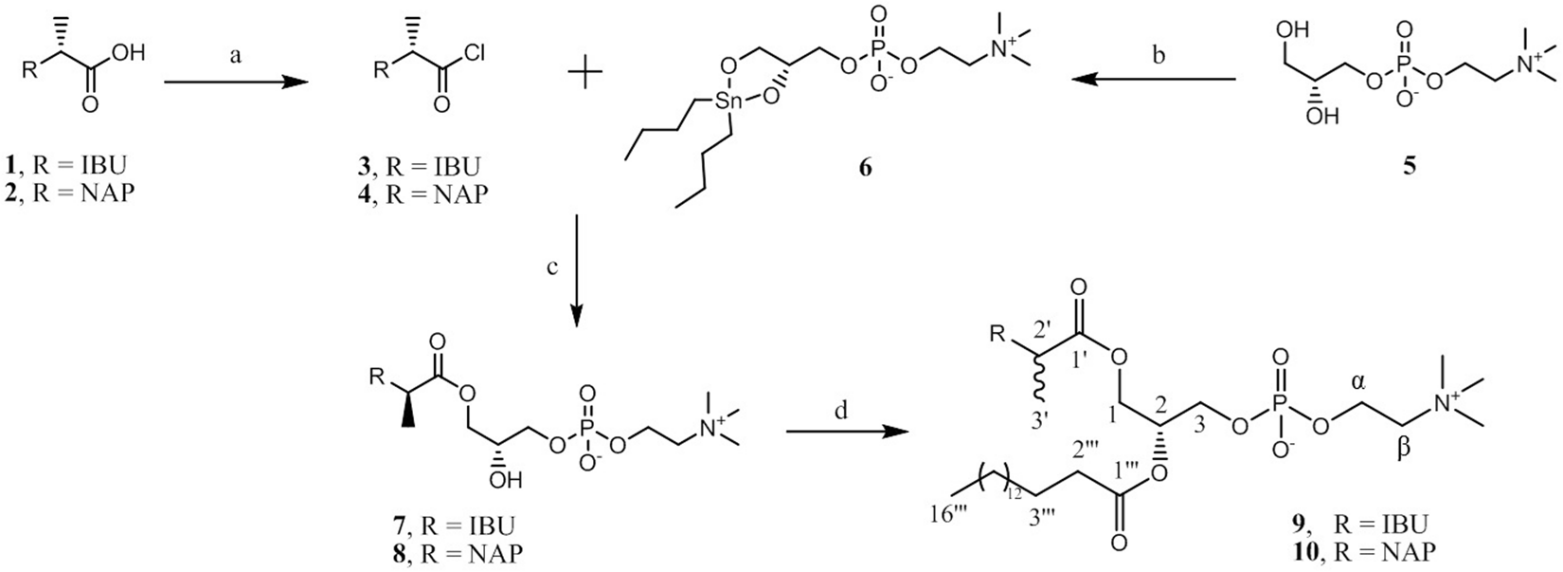
Synthesis of 2-lysophosphatidylcholines 7 and 8, and phosphatidylcholines 9 and 10. Reagents and conditions: (**a**) oxalyl chloride (3 equiv), DMF, CH_2_Cl_2_, 2 h; (**b**) DBTO, 2-propanol, reflux, 2 h; (**c**) TEA, 30 min; (**d**) palmitic acid, DMAP, DCC, 72 h.

Phosphatidylcholines containing ibuprofen (**1**) or naproxen (**2**) moieties at the *sn*-1 position (**7**–**10**) were synthesized according to Fig. 2. In the first steps, the corresponding chlorides **3** and **4**, and stannylene acetal (**6**) were obtained. Acyl chlorides resulted from the reaction of acids **1** and **2** with oxalyl chloride in the presence of catalytic amounts of anhydrous DMF. Then, according to known procedures^24,27,28^, the reaction of stannylene acetal (**6**) and acyl chloride (**3** or **4**) gave the corresponding 2-lysophosphatidylcholine (**7** or **8**) in good yields (68 and 70%, respectively). In the next step, lysophosphatidylcholines **7** and **8** were esterified with palmitic acid using the Steglich conditions to give phosphatidylcholines **9** and **10**, respectively.

The structures of lysophosphatidylcholines **7** and **8** were confirmed by NMR spectroscopy, since in the ^1^H and ^13^C NMR spectra of the two compounds, all hydrogen and carbon signals from the ibuprofen and naproxen moieties, respectively, were observed. Characteristic signals from the isobutyl group of compound **7** were observed at 0.85 and 0.86 ppm (d, *J* = 6.7 Hz) for the methyl groups and at 2.41 ppm (d, *J* = 7.2 Hz) for the methylene group. Moreover, the isobutyl methine proton was observed as a multiplet at δ = 1.81, CH_3_-3′ was detected as a doublet (*J* = 7.2 Hz) at 1.46 ppm, and the H-2′ methine proton was identified as a quartet (*J* = 7.2 Hz) at 3.72 ppm. Additionally, in the ^1^H NMR spectrum of lysophosphatidylcholine **8** a characteristic singlet of the methoxy group at δ = 3.88 was visible, and the H-2′ and H-2 methine protons overlapped and gave multiplets in the range of 3.89–3.93 ppm (Supplementary Information).

The introduction of palmitic acid into the *sn*-2 position was confirmed by the appearance of signals from fatty acid residue in the ^1^H NMR spectra of phosphatidylcholines **9** and **10**. Triplets from terminal methyl groups at δ = 0.84 (*J* = 7.1 Hz) for **9** and at δ = 0.85 (*J* = 7.0 Hz) for **10** were observed. Additionally, the structures of **9** and **10** were confirmed by the shift in the methine proton H-2 signals from δ = 3.92 (**7**) to δ = 5.19 (**9**) and from δ = 3.91 (**8**) to δ = 5.19 (**10**), respectively. Moreover, phosphatidylcholines **9** and **10** were obtained as mixtures of diastereomers, which was a result of the enolization of the carboester group with contribution of H-2′ from the ibuprofen or naproxen moiety under mildly alkaline conditions^29^. However, in acidic solutions, NSAIDs are reportedly highly stable and do not undergo racemization^30^.

The presence of two diastereomers of phosphocholine **9** was confirmed by the two quartets (*J* = 7.2 Hz) of H-2′ observed at 3.66 and 3.69 ppm and the two doublets (*J* = 7.2 Hz) of CH_3_-3′ at 1.43 and 1.44 ppm in the ^1^H NMR spectrum. Double signals were also observed for CH_2_-1 and CH_2_-3, and in the ^13^C NMR spectrum: C-1, C-1′, C-2′, C-3′, C-1″′, C-2″′, and C-3″′. The composition of the phosphatidylcholine isomers mixture (**9**) was determined by integrating one of the CH_2_-1 protons. Assuming that there was a higher content of phosphatidylcholine with *S-*ibuprofen, which was the starting enantiomer in the synthesis, the quantitative composition was defined as 77% 2 *R*,2′*S* isomer and 23% 2 *R*,2′*R* isomer.

Phosphatidylcholine (**10**) with a naproxen moiety at the *sn*-1 position and palmitoyl residue at the *sn*-2 position was also obtained as a mixture of diastereomers. However, a pronounced advantage of the 2 *R*,2′*S* isomer was observed in this case. The intensities of the ^1^H NMR signals from the 2 *R*,2′*R* isomer was so low (below 5%) that the percentage composition of the mixture could not be accurately determined. The smaller degree of racemization of the *S*-naproxen can be explained by the electron-donating effect of the methoxy group on the enol formation process.

**Figure 3:**
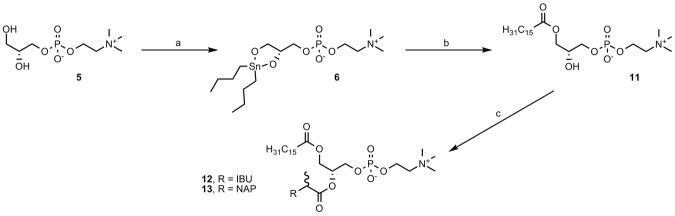
Synthesis of phosphatidylcholines 12 and 13. Reagents and conditions: room temperature (**a**) DBTO, 2-propanol, reflux, 2 h; (**b**) palmitoyl chloride (2 equiv), TEA, 30 min; (**c**) *S*-ibuprofen or *S*-naproxen (2 equiv), DMAP, DCC, 72 h.

Phosphatidylcholines containing palmitoyl residue at the *sn*-1 position and ibuprofen (**12**) or naproxen (**13**) at the *sn*-2 position were synthesized as depicted in Fig. 3. First, 2-lysophosphatidylcholine **11** was prepared according to a known procedure by reacting stannylene acetal (**6**) and palmitoyl chloride^31^. Then, **2**–lysophosphatidylcholine **11** was esterified with ibuprofen (**1**) or naproxen (**2**) using the Steglich conditions.

The structures of phosphatidylcholines **12** and **13** were confirmed by NMR spectroscopy. In the ^1^H NMR spectrum of **12** all signals of the palmitoyl residue were visible. Signals from the isobutyl group were observed at 2.41 ppm (d, *J* = 7.2 Hz) for the methylene protons, at 1.81 ppm (multiplet) for the methine proton, and at 0.86 ppm (d, *J* = 6.7 Hz) for the methyl groups. The presence of a multiplet at 5.20 ppm for H-2 indicated the attachment of the ibuprofen molecule onto the *sn*-2 position. Also, in the range of 7.03–7.17 ppm multiplets of the ibuprofen aromatic protons were visible. In addition, the structure of phosphatidylcholine **13** was confirmed by the presence of the following signals: 1.53 ppm (d, *J* = 7.1 Hz) from CH_3_-3′, 3.88 ppm (singlet) from the methoxy group, 5.21 ppm (multiplet) from H-2 and multiplets in the range of 7.08–7.70 ppm from the aromatic protons (Supplementary Information).

Similar to compound **9**, phosphatidylcholine **12**, was obtained as a mixture of diastereomers with similar quantitative composition: 73% of the 2 *R*,2′*S* isomer and 27% of the 2 *R*,2′*R* isomer. The presence of two diastereomers of **12** was indicated by the doubled signals for C3′ (17.57 and 17.63 ppm), C-2″′ (33.25 and 33.52 ppm), and C-3″′ (24.14 and 24.77 ppm) in the ^13^C NMR spectrum. Interestingly, the rest of the carbon atoms from phosphatidylcholine **12** were present in the ^13^C NMR spectrum as single signals. In contrast to compound **12**, phosphatidylcholine **13** containing naproxen at the *sn*-2 position was detected as one isomer (2 *R*,2′*S*).

**Figure 4:**
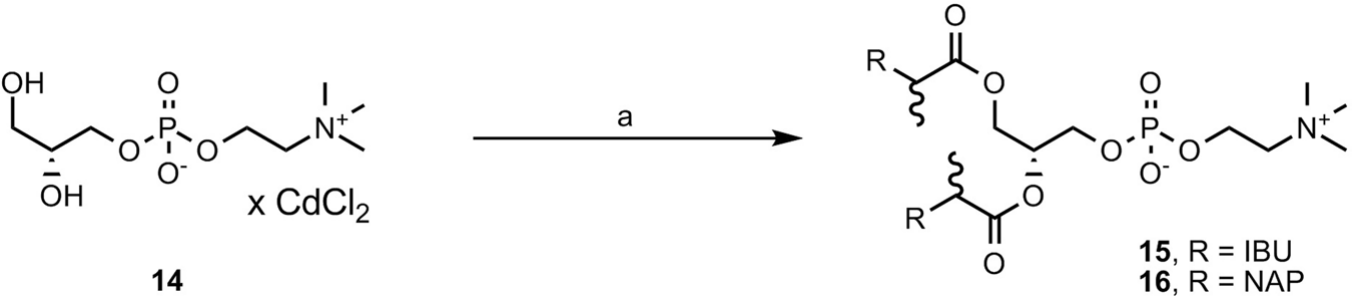
Synthesis of phosphatidylcholines 15 and 16. Reagents and conditions: (**a**) *S*-ibuprofen or *S*-naproxen (4 equiv), DMAP, DCC, 25 °C, N_2_, 48 h.

Phosphatidylcholines containing ibuprofen or naproxen moieties at both *sn*-1 and *sn*-2 positions (**15** and **16**) were synthesized in a one-step synthesis from the highly pure *sn*-glycero-3-phosphocholine (GPC) and ibuprofen (**1**) or naproxen (**2**), respectively (Fig. 4). In order to increase the solubility of GPC, its complex with cadmium chloride (**14**) was prepared according to a previously published procedure^24^. In the synthesis of **15** and **16**, the Steglich esterification of two free hydroxyl groups in the presence of a fourfold molar excess of ibuprofen (**1**) or naproxen (**2**) was applied.

**Figure 5:**
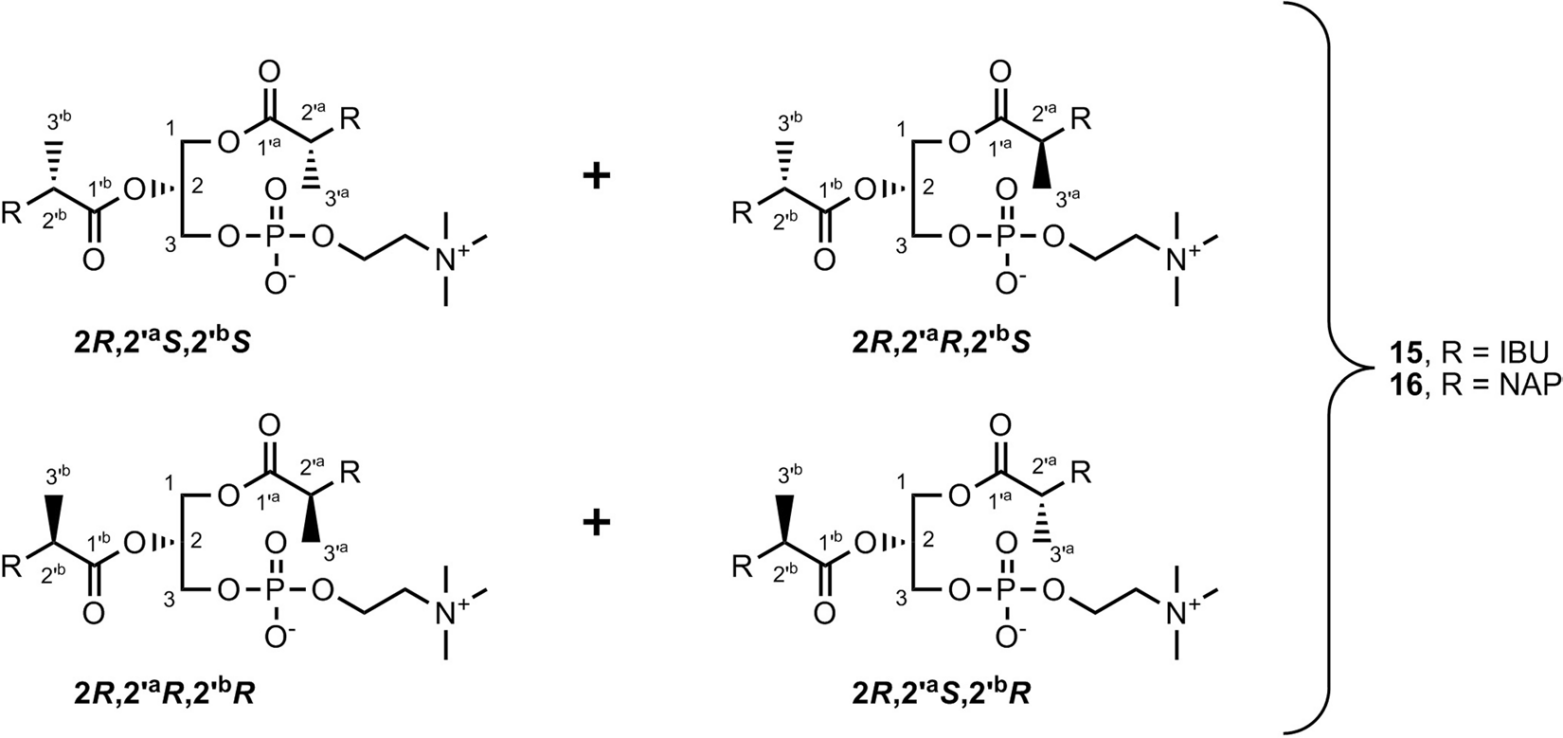
Diastereoisomers of phosphatidylcholines **15** and **16**. Indexes “^a^” and “^b^” are assigned to the acid residues at the *sn*-1 and *sn*-2 positions, respectively.

The use of mass spectrometry was highly significant in the determination of the structures of products **15** and **16**, since it detected intensive signals with *m/z* 634.3510 for **15** and *m/z* 682.2760 for **16**. Additionally, the structures of these compounds were confirmed by ^1^H, ^13^C, and ^31^P NMR spectroscopy. In the ^1^H NMR spectra of **15** and **16**, multiplied signals of acid moieties, glycerol, and choline were observed, which suggested the presence of four diastereomers in the product mixtures. Due to the substitution of identical molecules at the *sn*-1 and *sn*-2 positions and the racemization of acids bonded to GPC, the interpretation of the spectral data was complicated and very difficult. The racemization caused a formation of four diastereomers for both **15** and **16**, respectively (Fig. 5). Unfortunately, the separation of the individual isomers by a chiral HPLC column was impossible.

When looking at the ^1^H NMR spectrum of **15** in the range of 1.29–1.43 ppm, the overlapping doublets (*J* = 7.2 Hz) of CH_3_-3′^a-b^ could clearly be observed. Their differentiation was caused by the direct attachment of the methyl group to the chiral C-2′^a-b^ carbon atom of the ibuprofen moiety. Moreover, the methine protons of H-2′^a-b^ were detected as multiplets in the range of 3.43–3.69 ppm. The separation of signals was imperceptible due to the large distance from the ibuprofen isobutyl moiety to the stereogenic center. In addition, in the range of 0.84–0.88 ppm doublets (*J* = 6.6 Hz) from the isobutyl methyl groups were detected and at 3.11, 3.12, 3.15, and 3.16 ppm, four singlets from the choline methyl groups were detected. This indicated the formation of four diastereomers of compound **15**. Two-dimensional HSQC spectroscopy was instrumental in elucidating the structure of **15**. The measurements detected signals of the CH_2_-α (δ = 4.11 and 4.19) and CH_2_-β (δ = 3.49 and 3.55) protons, multiplets of the CH_2_-1 protons in the range of 3.98–4.42 ppm, and multiplets of the CH_2_-3 protons in the range of 3.84–3.96 ppm. Multiplets of the methine proton H-2 were detected at δ = 5.15 and 5.20 ppm. In the ^13^C NMR spectrum of phosphatidylcholine **15**, signals of C-3′^a-b^ and C-2′^a-b^ in the ranges of 17.44–17.87 and 44.24–44.58 ppm, respectively, were observed. Multiplied signals of the glycerophosphocholine carbon atoms were detected at 58.54 and 58.60 ppm (C-α, two d, *J*_C-P_ = 5.1 Hz), as well as in the ranges of 62.29–63.11 ppm (C-1 and C-3) and 65.75–65.90 (C-β). Interestingly, a single doublet (*J*_C-P_ = 8.0 Hz) from the chiral C-2 carbon atom appeared at 70.21 ppm. The carbon atoms from the aromatic ring did not show any differentiation with exception of C-1″^a-b^, which was closest to the chiral C-2′^a-b^ carbon atom and gave 8 signals in the range of 136.78–136.97 ppm. The signal of the ester carbon atom C-1′^a-b^ was also multiplied eight times and appeared in the range of 173.86–174.36 ppm. In the ^31^P NMR spectrum, only one signal from the phosphorus atom of **15** was visible at δ = –1.02.

In the synthesis of phosphatidylcholine **16**, a racemization of naproxen was also expected. The ^1^H NMR spectrum clearly indicated the formation of diastereomers and the multiplication of almost all signals. Signals of CH_3_-3′^a-b^ appeared as eight doublets (*J* = 7.2 Hz) in the range of 1.23–1.49 ppm, while H-2′^a-b^ were visible as overlapping quartets (*J* = 7.2 Hz) at 3.39–3.81 ppm. The signals of the methoxy group were observed at 3.86 and 3.87 ppm as two singlets. Hydrogen atoms from the glycerophosphocholine fragment were also distinctly differentiated. The CH_2_-1 groups of each diastereomer gave rise to two doublet of doublets (*J* = 12.0, 7.0 Hz) with signals at 3.98, 4.11, 4.15, and 4.16 ppm from one of the protons, and a doublet of doublets (*J* = 12.0, 3.0 Hz) with signals at 4.23, 4.33, 4.41, and 4.44 ppm from the second. The CH_2_-3 groups appeared as two multiplets in the range of 3.80–3.95 ppm. Moreover, at 2.90, 2.91, 3.01, and 3.03 ppm four singlets that were assigned to the choline methyl groups were detected. Particularly interesting was the chemical shift of the first two singlets, because these proton signals have never been observed at such a high field for phosphatidylcholines. The CH_2_-β methylene groups appeared on the spectrum as two multiplets at 3.08 and 3.36 ppm, and the CH_2_-α protons appeared as three multiplets in the range of 3.72–3.84 ppm and at 4.05 ppm. The shifts of the N(CH_3_)_3_ and CH_2_-β signals toward the higher field, as well as the differentiation of the CH_2_-α protons, may indicate the influence of the naproxen moiety from the *sn*-2 position on the hydrophilic choline part of the molecule. Modeling of the phosphatidylcholine **16** structure showed that this may be the result of the rotation around the C2-C3 bond and the existence of two distinctive rotamers: one with the phosphocholine moiety in *syn* conformation to the naproxen at the *sn*-2 position and the second one in the *anti*. In the first conformation, which was stabilized by the nucleophilic interaction of the –N^+^(CH_3_)_3_ group and naphthalene electrons, the choline moiety was located in the shielding cone of the magnetic field induced by the electrons of the naphthalene ring. In the second conformation, the electrons of the aromatic ring did not affect the chemical shifts of the hydrogen atoms of the choline moiety.

In the ^13^C NMR spectrum of phosphatidylcholine **16**, a differentiation of the signals of C-3′^a-b^ (17.22–17.66 ppm) and C-2′^a-b^ (44.42–44.83 ppm) was observed. A doubling of the signals from the carbon atoms was observed: C-α at 58.35 (d, *J*_C-P_ = 5.0 Hz) and at 58.59 ppm (d, *J*_C-P_ = 4.9 Hz), C-β in the range of 65.41–65.77 ppm (two m), and C-3 at 62.96 and 63.03 ppm (two d, *J*_C-P_ = 5.2 Hz) and at 63.13 and 63.18 ppm (two d, *J*_C-P_ = 5.6 Hz). In the range of 62.13–62.42 ppm, differentiated signals of the C-1 carbon atom were detected. Signals of the C-1′^a-b^ carbonyl atoms were visible at: 173.73, 173.82, 173.92, 174.09, 174.11, 174.21, and 174.25 ppm. The phosphorus atom was also differentiated, and the signals were registered at −1.01 and −1.19 ppm.

### Cytotoxicity of the phosphatidylcholines containing ibuprofen and naproxen moieties

Taking into consideration the idea of the future application of novel phosphatidylcholines containing ibuprofen or naproxen moieties as anti-inflammatory drugs with reduced gastrointestinal toxicity, it is necessary to analyze the cytotoxicity of these derivatives against the sensitive and normal cells. Then, the cytotoxicity of ibuprofen, naproxen, and their phospholipid derivatives was analyzed by a WST-1 cell proliferation assay. Our earlier studies indicated that among different cell lines, human promyelocytic leukemia HL-60 cells are the most sensitive to modified phosphatidylcholines^24,25,32^. Therefore, HL-60 cells, gastrointestinal origin - human colon cancer Caco-2 cells and normal porcine epithelial intestinal IPEC-J2 cells were used in these studies. Phosphatidylcholine derivatives were dissolved in dimethyl sulfoxide (DMSO) prior to examination. Unfortunately, due to the poor solubility of compound **13** in DMSO, its cytotoxicity was not tested. The solvent did not show cytotoxicity against analyzed cell lines.

**Figure 6:**
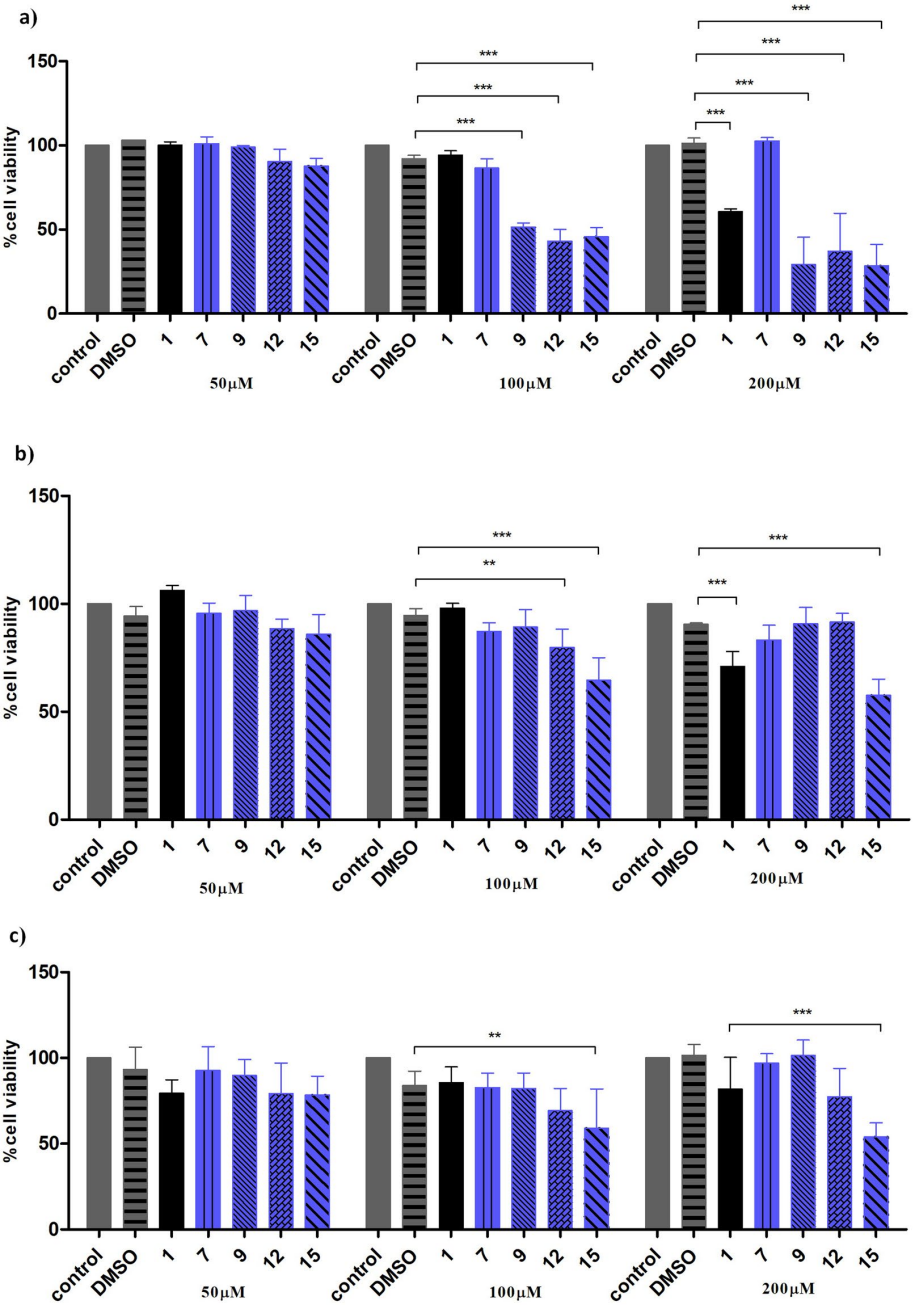
Viability of HL-60 cells (**a**) Caco-2 cells (**b**) and IPEC-J2 cells (**c**) grown in the presence of increased concentrations of ibuprofen (**1**) and phosphatidylcholines that contain ibuprofen in various *sn* positions (**7**, **9**, **12** and **15**). Percentage of viable cells was determined by the WST-1 assay, p < 0.01**, p < 0.001*** as described in Materials and Methods. Control – cells growing in appropriate complete media; DMSO – cells growing in the presence of DMSO.

In the case of human leukemic HL-60 cells, the experiments revealed that ibuprofen (**1**) was non-toxic up to concentration of 100 µM. In contrast to ibuprofen (**1**), 2-lysophosphatidylcholine **7** was non-toxic even at concentration 200 µM (Fig. 6a, Table 1). However, phosphatidylcholines **9**, **12** and **15** showed higher cytotoxic activity already at concentration 100 µM. The results of cytotoxicity studies of derivatives of ibuprofen against human colon carcinoma Caco-2 cells indicated that compounds **7**, **9** and **12** at 200 µM concentration were less toxic than ibuprofen itself (Fig. 6b, Table 1). Phosphatidylcholines containing ibuprofen moieties at both *sn*-1 and *sn*-2 positions (**15**) showed higher toxicity in comparison to **1**. Analysis of results of cytotoxic activity against normal porcine IPEC-J2 cells showed that compounds **7** and **9** were less toxic than ibuprofen (**1**) (Fig. 6c, Table 1). Phosphatidylcholines with ibuprofen in *sn*-2 position (**12**) and with two residues of ibuprofen (**15**) were more cytotoxic.

**Table 1:** IC_50_ values (±SD) of ibuprofen (**1**) and phosphatidylcholines containing ibuprofen moieties (**7**, **9**, **12** and **15**).

Compound	Position in PC^a^		IC_50_ [µM]^b^		
	*sn*-1	*sn*-2	HL-60	Caco-2	IPEC-J2
**1**	—	—	106.32 ± 17.45	114.43 ± 10.38	154.53 ± 7.57
**7**	IBU	OH	na	121.76 ± 2.76	na
**9**	IBU	PA	67.14 ± 10.92	125.23 ± 3.87	na
**12**	PA	IBU	77.42 ± 18.23	134.87 ± 18.16	79.23 ± 7.86
**15**	IBU	IBU	64.51 ± 10.44	81.32 ± 17.28	83.42 ± 1.51

na – not active in the range of concentrations tested (50–200 µM).

^a^IBU – ibuprofen.

^b^*IC*_50_ – half-maximal inhibitory concentration.

**Figure 7:**
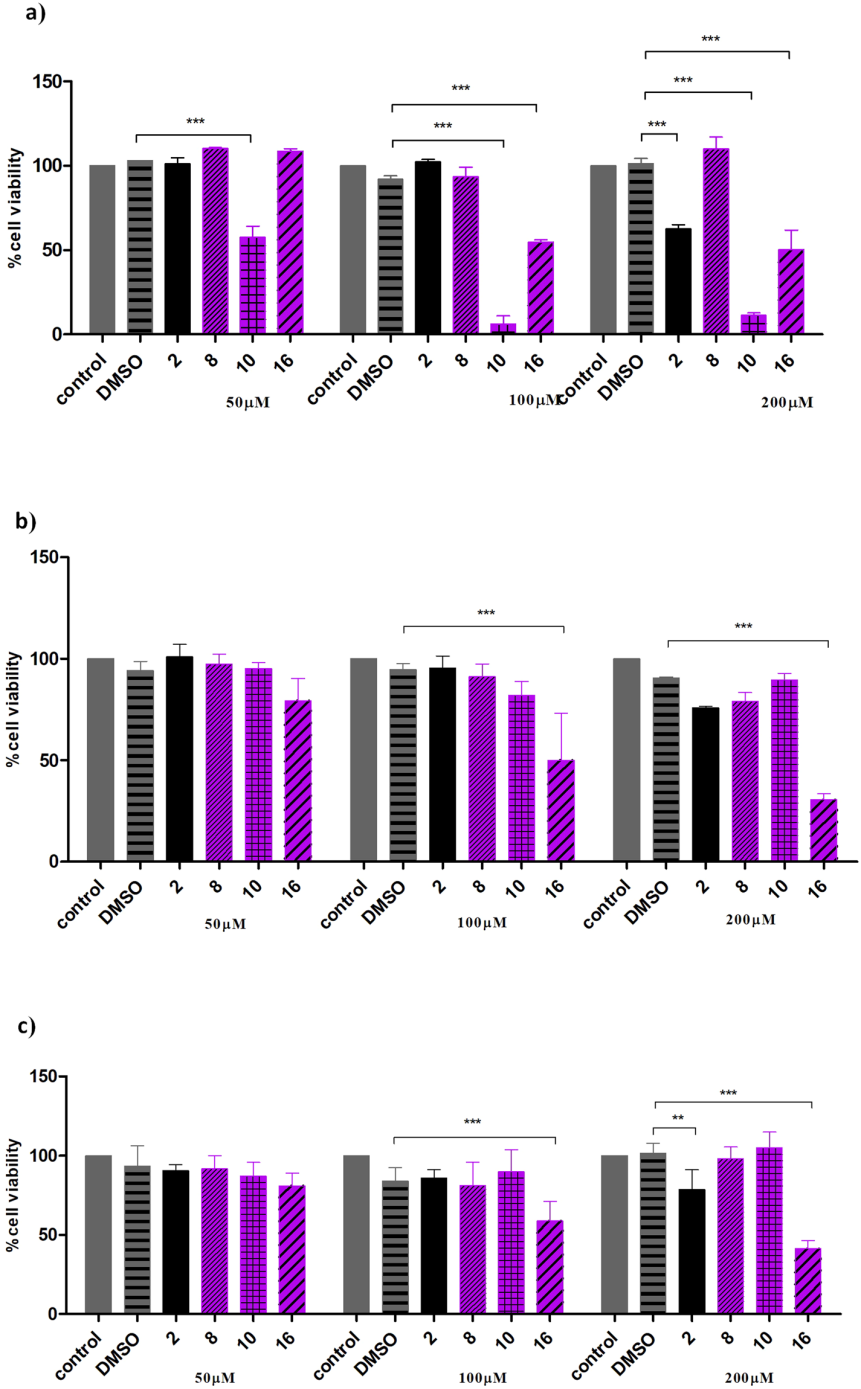
Viability of HL-60 cells (**a**) Caco-2 cells (**b**) and IPEC-J2 cells (**c**) grown in the presence of increased concentration of naproxen (**2**) and phosphatidylcholines that contain naproxen in various *sn* positions (**8, 10** and **16**). Percentage of viable cells was determined by the WST-1 assay, p < 0.01**, p < 0.001*** as described in Materials and Methods. Control – cells growing in appropriate complete media; DMSO – cells growing in the presence of DMSO.

Incubation of HL-60 cells revealed that naproxen (**2**) was non-toxic toward these cells up to concentration 100 µM. Among phosphatidylcholines containing naproxen, only compound **8** was significantly less cytotoxic (up to concentration of 200 µM) in comparison to free naproxen (Fig. 7a, Table 2). On the other hand, compound **16**, and especially **10** were highly toxic for HL-60 cells. In the case of colon carcinoma Caco-2 cells naproxen (**2**) was less toxic in comparison to leukemic HL-60 cells. Interestingly, also in contrast to leukemic cells, compound **10** was non-toxic up to concentration of 200 µM against the colon carcinoma cells. These cells were only sensitive to compound **16** (Fig. 7b, Table 2). The activity of phosphatidylcholines **8**, **10** and **16** have similar effect for both normal porcine IPEC-2 cells and colon carcinoma Caco-2 cells (Fig. 7b,c, Table 2). Phosphatidylcholines **8** and **10** were less toxic than naproxen (**2**) towards these cell lines at concentration 200 µM.

**Table 2:** IC_50_ values ( ± SD) of naproxen (**2**) and phosphatidylcholines containing naproxen moieties (**8**, **10** and **16**).

Compound	Position in PC^a^		IC_50_ [µM]^b^		
	*sn*-1	*sn*-2	HL-60	Caco-2	IPEC-J2
**2**	—	—	140.51 ± 8.51	112.76 ± 28.57	78.63 ± 15.86
**8**	NAP	OH	na	115.28 ± 26.12	na
**10**	NAP	PA	27.13 ± 5.13	na	na
**16**	NAP	IBU	80.11 ± 10.87	68.67 ± 18.43	58.43 ± 5.67

na – not active in the range of concentrations tested (50–200 µM).

^a^NAP *–* naproxen.

^*b*^*IC*_*50*_ *–* half-maximal inhibitory concentration.

The results of these studies showed that phosphatidylcholines containing NSAIDs at both *sn*-1 and *sn*-2 positions (**15** and **16**) were more toxic than ibuprofen or naproxen themselves. 2-Lysophosphatidylcholines (**7** and **8**) were less toxic against all tested cell lines. Phospholipids with NSAIDs in *sn*-1 and palmitic acid in *sn*-2 (**9** and **10**) were also less toxic against Caco-2 and normal cells (IPEC-J2).

## Conclusions

In this study, we synthesized eight phosphatidylcholines with ibuprofen or naproxen in their *sn*-1, *sn*-2 or *sn*-1 and *sn*-2 positions, and the cytotoxicity of the resulting compounds towards two cancer cells (HL-60 and Caco-2) and normal IPEC-J2 cells was evaluated. The results from the biological studies indicated that 2-lysophosphatidylcholines with NSAIDs at *sn*-1 position (**7** and **8**) and phosphatidylcholines with NSAIDs at *sn*-1 and palmitic acid residue at *sn*-2 (**9** and **10**) were less toxic against Caco-2 and normal cells (IPEC-J2). Compounds **7** and **8** were also less toxic than ibuprofen or naproxen against HL-60 cells. These results confirmed our earlier observations that phospholipid derivatives of active compounds are less cytotoxic than the compounds themselves. These data indicate also that phospholipids could be used as carriers of NSAIDs. However, further biological studies are necessary in order to confirm this proposal.

## Materials and Methods

### Chemicals and Analysis Conditions

*sn*-Glycero-3-phosphocholine (GPC) was purchased from Bachem (Bubendorf, Switzerland). *S*-Ibuprofen (**1**), *S*-naproxen (**2**), *N,N′*-dicyclohexylcarbodiimide (DCC), 4-(*N,N*-dimethylamino)pyridine (DMAP), dibutyltin(IV) oxide (DBTO), triethylamine (TEA), palmitoyl chloride, oxalyl chloride, Dowex^®^ 50WX8 H^+^ form (an ion-exchange resin), hexane, methanol, 2-propanol for liquid chromatography, and chloroform (CHROMASOLV^®^) were purchased from Sigma-Aldrich (Munich, Germany). Chloroform and methanol for column and thin-layer chromatography (TLC) were purchased from Stanlab (Lublin, Poland). TLC was performed on Merck Kieselgel 60 F_254_ plates (0.2 mm silica gel with fluorescent indicator UV_254_). As a visualization reagent, primuline spray (0.05% in acetone:water, 80:20, v/v) was used. Visualization was determined using UV light (λ = 365 nm). Column chromatography was performed on silica gel (Kieselgel 60, 0.040–0.063 mm, 230–400 mesh ASTM; Merck, Darmstadt, Germany) using an eluent mixture of CHCl_3_:CH_3_OH:H_2_O (65:25:4, v/v/v). The purity of all final phosphatidylcholines was determined by HPLC to be 96% or higher. HPLC was performed on an UltiMate 3000 apparatus (Dionex, Sunnyvale, CA, USA) with a charged aerosol detector (Corona CAD, ESA Biosciences, Chelmsford, USA). Nitrogen was used as a nebulizing gas at a pressure of 35 psi and at an acquisition range of 100 pA. A BETASIL Diol column (150 × 4.6 mm, 5 µm; Thermo Scientific, MA, USA) was used. The mobile phase was set at a flow rate of 1.5 mL/min and consisted of the following solvents: (A) water, (B) hexane, and (C) 2-propanol. The mobile phase was run as %A:%B:%C (v/v/v) using the following gradient timetable: 0 min - 0:43:57, 5 min -3:40:57, 8 min - 10:40:50, 13 min - 10:40:50, 13.1 min - 0:43:57, and 22 min - 0:43:57. All NMR spectra were recorded on an Avance II 600 MHz spectrometer (Brüker, Billerica, MA, USA) working at a frequency of 600 MHz for ^1^H, 150 MHz for ^13^C, and 243 MHz for ^31^P. Samples of all compounds were measured in a mixture of CDCl_3_:CD_3_OD (2:1, v/v). The chemical shifts were calibrated using: the methanol proton signals (δ_H_ = 3.31) in the ^1^H NMR spectra and the CDCl_3_ carbon atoms (δ_c_ = 77.0) in the ^13^C NMR spectra. H_3_PO_4_ (85%) was used as an external standard for the ^31^P NMR chemical shifts. HRMS spectra were recorded using the ESI technique on a spectrometer (ESI-Q-TOF Premier XE; Waters, Milford, MA, USA). Melting points (MPs, uncorrected) were determined on a Boetius apparatus.

The complexes of cadmium chloride and *sn*-glycero-3-phosphocholine (**14**, GPCxCdCl_2_), and 1-palmitoyl-2-hydroxy-*sn*-glycero-3-phosphocholine (**11**) were prepared according to previously reported procedures^31,33^.

### Syntheses

General procedure for the preparation of 2-lysophosphatidylcholines **7** and **8**. *S*-Ibuprofen (**1**, 0.3 g, 1.46 mmol) or *S*-naproxen (**2**, 0.34 g, 1.46 mmol) was dissolved in anhydrous methylene chloride (5 mL), and catalytic amount (2 drops) of anhydrous DMF was added to the mixture. Excess of oxalyl chloride (3 equiv, 376 μL, 4.38 mmol) was added dropwise to the stirring solution. The reaction was carried out at room temperature for 2 h and the solvent and excess of oxalyl chloride were subsequently evaporated. The resulting chloride **3** or **4** was immediately used for the synthesis of 2-lysophosphatidylcholine **7** or **8**.

GPC (0.25 g, 0.97 mmol) and DBTO (0.242 g, 0.97 mmol) were suspended in 15 mL of 2-propanol and refluxed for 2 h. The mixture was cooled to room temperature and TEA (0.203 mL, 1.46 mmol) and corresponding chloride **3** or **4** were then added to it. The mixture was stirred for 30 min. After completion of the reaction (checked by TLC) the solvent was evaporated under reduced pressure and the residue was purified by silica gel column chromatography using chloroform:methanol:water (65:25:2 → 65:25:4) to give the corresponding 2-lysophosphatidylcholine (**7** or **8**).

1-[2′S-(4″-Isobutylphenyl)]propanoyl-2-hydroxy-sn-glycero-3-phosphocholine (**7**): Colorless, waxy product; yield 68% (0.292 g). ^1^H NMR (600 MHz, CDCl_3_:CD_3_OD, 2:1, v/v) δ: 0.86 (d, *J* = 6.7 Hz, 6 H, (C**H**_**3**_)_**2**_-CH-CH_2_-), 1.46 (d, *J* = 7.2 Hz, 3 H, CH_3_-3′), 1.81 (m, 1 H, (CH_3_)_2_-C**H**-CH_2_-), 2.41 (d, *J* = 7.2 Hz, 2 H, (CH_3_)_2_-CH-C**H**_**2**_-), 3.17 (s, 9 H, N(CH_3_)_3_), 3.58 (m, 2 H, CH_2_-β), 3.72 (q, *J* = 7.2 Hz, 1 H, H-2′), 3.81 (ddd, *J* = 11.0, 7.5, 5.7 Hz, 1 H, one of CH_2_-3), 3.88 (m, 1 H, one of CH_2_-3), 3.92 (m, 1 H, H-2), 4.02 (dd, *J* = 11.5, 6.1 Hz, 1 H, one of CH_2_-1), 4.18 (dd, *J* = 11.5, 5.0 Hz, 1 H, one of CH_2_-1), 4.23 (m, 2 H, CH_2_-α), 7.04–7.08 (m, 2 H, H-2″ and H-6″), 7.14–7.18 (m, 2 H, H-3″ and H-5″). ^13^C NMR (150 MHz, CDCl_3_:CD_3_OD, 2:1, v/v) δ: 17.85 (C-3′), 21.63 ((CH_3_)_2_-CH-CH_2_-), 29.68 ((CH_3_)_2_-CH-CH_2_-), 44.46 (C-2′), 44.53 ((CH_3_)_2_-CH-CH_2_-), 53.48, 53.51 and 53.53 (N(CH_3_)_3_), 58.67 (d, *J*_C-P_ = 4.7 Hz, C-α), 64.76 (C-1), 65.87 (C-β), 66.34 (d, *J*_C-P_ = 5.6 Hz, C-3), 68.11 (d, *J*_C-P_ = 6.6 Hz, C-2), 126.62 (C-3″ and C-5″), 128.82 (C-2″ and C-6″), 137.06 (C-1″), 140.15 (C-4″), 174.80 (C-1′). ^31^P NMR (243 MHz, CDCl_3_:CD_3_OD, 2:1, v/v) δ: –0.34. ESI-MS: *m/z* calculated for C_21_H_36_NO_7_P [M + H]^+^: 446.2308. Found: 446.2319.

1-[2′S-(6″-Methoxynaphthalenyl)]propanoyl-2-hydroxy-sn-glycero-3-phosphocholine (**8**): Light pink powder; yield 70% (0.318 g); mp 162–164 °C. ^1^H NMR (600 MHz, CDCl_3_:CD_3_OD, 2:1, v/v) δ: 1.54 (d, *J* = 7.2 Hz, 3 H, CH_3_-3′), 3.08 (s, 9 H, N(CH_3_)_3_), 3.44 (m, 2 H, CH_2_-β), 3.79 (m, 1 H, one of CH_2_-3), 3.85 (m, 1 H, one of CH_2_-3), 3.88 (s, 3 H, O-CH_3_), 3.89–3.93 (m, 2 H, H-2′ and H-2), 4.07 (dd, *J* = 11.4, 6.1 Hz, 1 H, one of CH_2_-1′), 4.13 (m, 2 H, CH_2_-α), 4.18 (dd, *J* = 11.4, 5.0 Hz, 1 H, one of CH_2_-1), 7.09–7.13 (m, 2 H, H-5″ and H-7″), 7.37 (m, 1 H, H-10″), 7.64 (s, 1 H, H-2″), 7.67–7.70 (m, 2 H, H-4″ and H-9″). ^13^C NMR (150 MHz, CDCl_3_:CD_3_OD, 2:1, v/v) δ: 17.84 (C-3′), 44.85 (C-2′), 53.41, 53.44 and 53.46 (N(CH_3_)_3_), 54.68 (O-CH_3_), 58.55 (d, *J*_C-P_ = 5.0 Hz, C-α), 64.75 (C-1), 65.80 (C-β), 66.24 (d, *J*_C-P_ = 5.7 Hz, C-3), 68.06 (d, *J*_C-P_ = 6.7 Hz, C-2), 105.14 (C-7″), 118.47 (C-5″), 125.41 (C-2″), 125.72 (C-10″), 126.72 and 128.77 (C-4″ and C-9″), 128.48 (C-3″), 133.30 (C-8″), 135.12 (C-1″), 157.24 (C-6″), 174.63 (C-1′). ^31^P NMR (243 MHz, CDCl_3_:CD_3_OD, 2:1, v/v) δ: –0.30. ESI-MS: *m/z* calculated for C_22_H_32_NO_8_P [M + H]^+^: 470.1944. Found: 470.1961.

General procedure for the preparation of phosphatidylcholines **9** and **10**. Lysophosphatidylcholine **7** (0.089 g, 0.2 mmol) or **8** (0.094 g, 0.2 mmol) and DMAP (0.025 g, 0.2 mmol) were dissolved in dry methylene chloride (5 mL). The, a mixture of palmitic acid (2 equiv, 0.103 g, 0.4 mmol) and DCC (3 equiv, 0.124 g, 0.6 mmol) in the same solvent (5 mL) was added. The reaction was stirred for 72 h at room temperature. The suspension was filtered off and stirred with Dowex^®^ resin (50WX8 H^+^) for 30 min. The resin was then filtered off and washed with a Folch mixture (15 mL). The solvent was evaporated and the crude product was purified by column chromatography on silica gel (CHCl_3_:CH_3_OH:H_2_O, 65:25:4) to give the corresponding phosphatidylcholines **9** or **10**. The yields and the physical and spectroscopic data of the products are given below.

1-[2′-(4″-Isobutylphenyl)]propanoyl-2-palmitoyl-sn-glycero-3-phosphocholine (**9**): Colorless waxy product; yield 25% (0.034 g). ^1^H NMR (600 MHz, CDCl_3_:CD_3_OD, 2:1, v/v) δ: 0.84 (t, *J* = 7.1 Hz, 3 H, CH_3_-16″′), 0.86 (d, *J* = 6.6 Hz, 6 H, (C**H**_**3**_)_2_-CH-CH_2_-), 1.22–1.26 (m, 24 H, CH_2_-4″′-CH_2_-15″′), 1.43 and 1.44 (two d, *J* = 7.2 Hz, 3 H, CH_3_-3′), 1.47–1.55 (m, 2 H, CH_2_-3″′), 1.80 (m, 1 H, (CH_3_)_2_-C**H**-CH_2_-), 2.15 and 2.20 (two dt, *J* = 16.0, 7.6 Hz, 2 H, CH_2_-2″′), 2.40 (d, *J* = 7.2 Hz, 2 H, (CH_3_)_2_-CH-C**H**_**2**_-), 3.16 (s, 9 H, N(CH_3_)_3_), 3.55 (m, 2 H, CH_2_-β), 3.66 and 3.69 (two q, *J* = 7.2 Hz, 1 H, H-2′), 3.91–3.99 (two m, 2 H, CH_2_-3), 4.03 and 4.09 (two dd, *J* = 12.0, 7.3 Hz, 1 H, one of CH_2_-1), 4.20 (m, 2 H, CH_2_-α), 4.25 and 4.38 (two dd, *J* = 12.0, 3.1 Hz, 1 H, one of CH_2_-1), 5.19 (m, 1 H, H-2), 7.03–7.06 (m, 2 H, H-2″ and H-6″), 7.11–7.15 (m, 2 H, H-3″ and H-5″). ^13^C NMR (150 MHz, CDCl_3_:CD_3_OD, 2:1, v/v) δ: 13.44 (C-16″′), 17.76 (C-3′), 21.73 ((CH_3_)_2_-CH-CH_2_-), 24.36 (C-3″′), 28.68–31.46 (C-4″′-C-15″′), 29.76 ((CH_3_)_2_-CH-CH_2_-), 33.63 (C-2″′), 44.57 (C-2′), 44.59 ((CH_3_)_2_-CH-CH_2_-), 53.58, 53.60 and 53.63 (N(CH_3_)_3_), 58.67 (d, *J*_C-P_ = 4.6 Hz, C-α), 62.65 (C-1), 63.28 (d, *J*_C-P_ = 4.9 Hz, C-3), 65.99 (C-β), 69.79 (d, *J*_C-P_ = 7.6 Hz, C-2), 126.68 (C-3″ and C-5″), 128.88 (C-2″ and C-6″), 136.94 (C-1″), 140.24 (C-4″), 173.10 (C-1″′), 174.43 (C-1′). ^31^P NMR (243 MHz, CDCl_3_:CD_3_OD, 2:1, v/v) δ: –0.76. ESI-MS: *m/z* calculated for C_37_H_66_NO_8_P [M + H]^+^: 684.4604. Found: 684.4619.

1-[2′-(6″-Methoxynaphthalenyl)]propanoyl-2-palmitoyl-sn-glycero-3-phosphocholine (**10**): Colorless waxy product; yield 57% (0.081 g). ^1^H NMR (600 MHz, CDCl_3_:CD_3_OD, 2:1, v/v) δ: 0.85 (t, *J* = 7.0 Hz, 3 H, CH_3_-16″′), 1.18–1.27 (m, 24 H, CH_2_-4″′-CH_2_-15″′), 1.35–1.41 (m, 2 H, CH_2_-3″′), 1.53 (d, *J* = 7.2 Hz, 3 H, CH_3_-3′), 1.98 (dt, *J* = 15.6, 7.6 Hz, 1 H, one of CH_2_-2″′), 2.07 (dt, *J* = 15.6, 7.6 Hz,1 H, one of CH_2_-2″′), 3.12 (s, 9 H, N(CH_3_)_3_), 3.47 (m, 2 H, CH_2_-β), 3.84 (q, *J* = 7.2 Hz, 1 H, H-2′), 3.88 (s, 3 H, O-CH_3_), 3.90–3.93 (m, 2 H, CH_2_-3), 4.14 (m, 2 H, CH_2_-α), 4.16 (dd, *J* = 12.0, 7.1 Hz, 1 H, one of CH_2_-1), 4.38 (dd, *J* = 12.0, 3.1 Hz, 1 H, one of CH_2_-1), 5.19 (m, 1 H, H-2), 7.09–7.12 (m, 2 H, H-5″ and H-7″), 7.34 (m, 1 H, H-10″), 7.61 (s, 1 H, H-2″), 7.66–7.69 (m, 2 H, H-4″ and H-9″). ^13^C NMR (150 MHz, CDCl_3_:CD_3_OD, 2:1, v/v) δ: 13.33 (C-16″′), 17.63 (C-3′), 24.17 (C-3″′), 22.12–31.39 (C-4″′-C-15″′), 33.43 (C-2″′), 44.83 (C-2′), 53.41, 53.43 and 53.46 (N(CH_3_)_3_), 54.61 (O-CH_3_), 58.54 (d, *J*_C-P_ = 4.9 Hz, C-α), 62.60 (C-1), 63.09 (d, *J*_C-P_ = 5.3 Hz, C-3), 65.82 (C-β), 69.64 (d, *J*_C-P_ = 7.8 Hz, C-2), 105.08 (C-7″), 118.50 (C-5″), 125.40 (C-2″), 125.62 (C-10″), 126.69 and 128.72 (C-4″ and C-9″), 128.46 (C-3″), 133.30 (C-8″), 134.89 (C-1″), 157.25 (C-6″), 173.00 (C-1″′), 174.27 (C-1′). ^31^P NMR (243 MHz, CDCl_3_:CD_3_OD, 2:1, v/v) δ: –0.82. ESI-MS: *m/z* calculated for C_38_H_62_NO_9_P [M + H]^+^: 708.4240. Found: 708.4233.

General procedure for the preparation of phosphatidylcholines **12** and **13**. To a solution of PLPC (**11**, 0.2 g, 0.4 mmol) and DMAP (0.049 g, 0.4 mmol) in anhydrous methylene chloride (5 mL), a mixture of *S*-ibuprofen (**1**, 0.163 g, 0.8 mmol) or *S*-naproxen (**2**, 0.184 g, 0.8 mmol) and DCC (0.165 g, 0.8 mmol) in the same solvent (3 mL) was added. The reaction was stirred for 72 h at room temperature. The resulting precipitate was filtered off and a Dowex^®^ 50WX8 H^+^ form was added. After 30 min of stirring, the resin was filtered off on a Shott funnel and washed with a Folch mixture (15 mL). Products **12** and **13** were separated by column chromatography (CHCl_3_:CH_3_OH:H_2_O, 65:25:4).

1-Palmitoyl-2-[2′-[4″-isobutylphenyl)]propanoyl-sn-glycero-3-phosphocholine (**12**): Colorless waxy product; yield 41% (0.113 g). ^1^H NMR (600 MHz, CDCl_3_:CD_3_OD, 2:1, v/v) δ: 0.85 (t, *J* = 7.1 Hz, 3 H, CH_3_-16″′), 0.86 (d, *J* = 6.6 Hz, 6 H, (C**H**_**3**_)_2_-CH-CH_2_-), 1.23–1.26 (m, 24 H, CH_2_-4″′-CH_2_-15″′), 1.45 (m, 1 H, one of CH_2_-3″′), 1.44 (d, *J* = 7.1 Hz, CH_3_-3′), 1.53 (m, 1 H, one of CH_2_-3″′), 1.81 (m, 1 H, (C**H**_3_)_2_-C**H**-CH_2_-), 2.02–2.31 (two m, 2 H, CH_2_-2″′), 2.41 (d, *J* = 7.2 Hz, 2 H, (CH_3_)_2_-CH-C**H**_**2**_-), 3.19 (s, 9 H, N(CH_3_)_3_), 3.52 and 3.58 (m, 2 H, CH_2_-β), 3.67 and 3.71 (two q, *J* = 7.1 Hz, 1 H, H-2′), 3.91–3.99 (two m, 2 H, CH_2_-3), 4.05 and 4.11 (two dd, *J* = 11.9, 7.5 Hz, 1 H, one of CH_2_-1), 4.22 (m, 2 H, CH_2_-α), 4.26 and 4.39 (two dd, *J* = 11.9, 3.1 Hz, 1 H, one of CH_2_-1), 5.20 (m, 1 H, H-2), 7.03–7.08 (m, 2 H, H-2″ and H-6″), 7.12–7.17 (m, 2 H, H-3″ and H-5″). ^13^C NMR (150 MHz, CDCl_3_:CD_3_OD, 2:1, v/v) δ: 13.29 (C-16″′), 17.57 (C-3″), 21.59 ((CH_3_)_2_-CH-CH_2_-), 24.14 (C-3″′), 28.57–31.37 (C-4″′-C-15″′), 29.68 ((CH_3_)_2_-CH-CH_2_-), 33.25 (C-2″′), 44.44 and 44.48 (C-2′ and (CH_3_)_2_-CH-CH_2_-), 53.44, 53.46 and 53.49 (N(CH_3_)_3_), 58.60 (C-α), 62.03 (C-1), 63.19 (d, *J*_C-P_ = 4.6 Hz, C-3), 65.86 (C-β), 70.21 (d, *J*_C-P_ = 6.3 Hz, C-2), 126.56 (C-3″ and C-5″), 128.75 (C-2″ and C-6″), 136.97 (C-1″), 140.07 (C-4″), 173.35 (C-1″′), 174.10 (C-1′). ^31^P NMR (243 MHz, CDCl_3_:CD_3_OD, 2:1, v/v) δ: –0.78. ESI-MS: *m/z* calculated for C_37_H_66_NO_8_P [M + H]^+^: 684.4604. Found: 684.4617.

1-Palmitoyl-2-[2′S-(6″-methoxynaphthalenyl)]propanoyl-sn-glycero-3-phosphocholine (**13**): Colorless waxy product; yield 53% (0.152 g). ^1^H NMR (600 MHz, CDCl_3_:CD_3_OD, 2:1, v/v) δ: 0.85 (t, *J* = 6.8 Hz, 3 H, CH_3_-16″′), 0.98–1.24 (multiplets, 24 H, CH_2_-4″′-CH_2_-15″′), 1.25–1.27 (m, 2 H, CH_2_-3″′), 1.53 (d, *J* = 7.1 Hz, 3 H, CH_3_-3′), 1.79 and 1.84 (two dt, *J* = 15.6, 7.6 Hz, 2 H, CH_2_-2″′), 3.13 (s, 9 H, N(CH_3_)_3_), 3.46 (m, 2 H, CH_2_-β), 3.85 (m, 1 H, H-2′), 3.88 (s, 3 H, O-CH_3_), 3.95–3.99 (m, 2 H, CH_2_-3), 4.06 (dd, *J* = 11.9, 7.5 Hz, 1 H, one of CH_2_-1), 4.13 (m, 2 H, CH_2_-α), 4.24 (dd, *J* = 11.9, 3.1 Hz, 1 H, one of CH_2_-1), 5.21 (m, 1 H, H-2), 7.08–7.12 (m, 2 H, H-5″ and H-7″), 7.36 (m, 1 H, H-10″), 7.63 (s, 1 H, H-2″), 7.65–7.69 (m, 2 H, H-4″ and H-9″). ^13^C NMR (150 MHz, CDCl_3_:CD_3_OD, 2:1, v/v) δ: 13.42 (C-16″′), 17.56 (C-3′), 24.04 (C-3″′), 22.16–31.48 (C-4″′-C-15″′), 33.14 (C-2″′), 44.88 (C-2′), 53.50, 53.52 and 53.55 (N(CH_3_)_3_), 54.68 (O-CH_3_), 58.52 (d, *J*_C-P_ = 4.9 Hz, C-α), 62.00 (C-1), 63.24 (d, *J*_C-P_ = 5.2 Hz, C-3), 65.87 (C-β), 70.44 (d, *J*_C-P_ = 7.8 Hz, C-2), 105.09 (C-7″), 118.54 (C-5″), 125.40 (C-2″), 125.72 (C-10″), 126.68 and 128.77 (C-4″ and C-9″), 128.48 (C-3″), 133.31 (C-8″), 134.99 (C-1″), 157.29 (C-6″), 173.34 and 173.99 (C-1′ and C-1″′). ^31^P NMR (243 MHz, CDCl_3_:CD_3_OD, 2:1, v/v) δ: –0.73. ESI-MS: *m/z* calculated for C_38_H_62_NO_9_P [M + H]^+^: 708.4240. Found: 708.4230.

General procedure for the synthesis of compounds **15** and **16**. The GPC × CdCl_2_ complex (**14**, 0.2 g, 0.46 mmol) and DMAP (0.056 g, 0.46 mmol) were dissolved in anhydrous methylene chloride (5 mL). To this solution, a solution of DCC (0.380 g, 1.84 mmol) and *S*-ibuprofen (**1**, 0.380 g, 1.84 mmol) or *S*-naproxen (**2**, 0.424 g, 1.84 mmol) in anhydrous methylene chloride (5 mL) was added. The mixture was stirred for 48 h at 25 °C under nitrogen atmosphere and the progress of the reaction was monitored by TLC. The precipitated dicyclohexylurea was then filtered off and a Dowex^®^ 50WX8 H^+^ form was added to the mixture. The solution was stirred for 30 min, after which the resin was filtered off on a Shott funnel. Then, the resin was washed with 15 mL of Folch mixture (CHCl_3_:CH_3_OH, 2:1) and the solvent was evaporated *in vacuo*. The residues were purified by column chromatography using a mixture of chloroform/methanol/water (65:25:4) as eluent to give a colorless waxy product **15** or **16**.

1,2-Di-[2′-(4″-isobutylphenyl)]propanoyl-sn-glycero-3-phosphocholine (**15**): Colorless waxy product; yield 61% (0.177 g). ^1^H NMR (600 MHz, CDCl_3_:CD_3_OD, 2:1, v/v) δ: 0.84–0.88 (doublets, *J* = 6.6 Hz, 12 H, (CH_3_)_2_-CH-CH_2_-), 1.29–1.43 (doublets, *J* = 7.2 Hz, 6 H, CH_3_-3′^a-b^), 1.76–1.85 (two m, 2 H, 2 × (CH_3_)_2_-CH-CH_2_-), 2.39–2.43 (m, 4 H, (CH_3_)_2_-CH-CH_2_-), 3.11, 3.12, 3.15 and 3.16 (four s, 9 H, N(CH_3_)_3_), 3.43–3.69 (multiplets, 2 H, H-2′^a-b^), 3.49 and 3.55 (two m, 2 H, CH_2_-β), 3.84–3.96 (two m, 2 H, CH_2_-3), 3.98, 4.08–4.29, 4.35 and 4.42 (multiplets, 2 H, CH_2_-1), 4.11 and 4.19 (two m, 2 H, CH_2_-α), 5.15 and 5.20 (two m, 1 H, H-2), 7.02–7.16 (two m, 8 H, H-2″^a-b^, H-3″^a-b^, H-5″^a-b^ and H-6″^a-b^). ^13^C NMR (150 MHz, CDCl_3_:CD_3_OD, 2:1, v/v) δ: 17.44, 17.51, 17.60, 17.66, 17.69, 17.78 and 17.87 (C-3′^a-b^), 21.58 ((CH_3_)_2_-CH-CH_2_-), 29.65 ((CH_3_)_2_-CH-CH_2_-), 44.24–44.58 (C-2′^a-b^ and (CH_3_)_2_-CH-CH_2_-), 53.45 and 53.47 (N(CH_3_)_3_), 58.54 and 58.60 (dwa d, *J*_C-P_ = 5.1 Hz,C-α), 62.29–63.11 (C-1 and C-3), 65.75–65.90 (C-β), 70.21 (d, *J*_C-P_ = 8.0 Hz, C-2), 126.58, 126.60, 126.62 and 126.66 (C-3″^a-b^ and C-5″^a-b^), 128.74, 128.76, 128.78 and 128.80 (C-2″^a-b^ and C-6″^a-b^), 136.78, 136.80, 136.84, 136.85, 136.89, 136.92, 136.95 and 136.97 (C-1″^a-b^), 140.06, 140.10, 140.13 and 140.15 (C-4″^a-b^), 173.86, 173.94, 173.97, 174.05, 174.23, 174.25, 174.33 and 174.36 (C-1′^a-b^). ^31^P NMR (243 MHz, CDCl_3_:CD_3_OD, 2:1, v/v) δ: –1.02. ESI-MS: *m/z* calculated for C_34_H_52_NO_8_P [M + H]^+^: 634.3509. Found: 634.3510.

1,2-Di-[2′-(6″-methoxynaphthalenyl)]propanoyl-sn-glycero-3-phosphocholine (**16**): Colorless waxy product; yield 72% (0.225 g). ^1^H NMR (600 MHz, CDCl_3_:CD_3_OD, 2:1, v/v) δ: 1.23, 1.29, 1.33, 1.35, 1.42, 1.44, 1.48 and 1.49 (eight d, *J* = 7.2 Hz, 6 H, CH_3_-3′^a-b^), 2.90, 2.91, 3.01 and 3.03 (four s, 9 H, N(CH_3_)_3_), 3.08 and 3.36 (two m, 2 H, CH_2_-β), 3.39, 3.41, 3.54, 3.56 and 3.72–3.81 (quartets, *J* = 7.2 Hz, 2 H, H-2′^a-b^), 3.72–3.84 and 4.05 (multiplets, 2 H, CH_2_-α), 3.80–3.95 (two m, 2 H, CH_2_-3), 3.86 and 3.87 (two s, 6 H, O-CH_3_), 3.98, 4.11, 4.15 and 4.16 (four dd, *J* = 12.0, 7.0 Hz, 1 H, one of CH_2_-1), 4.23, 4.33, 4.41 and 4.44 (four dd, *J* = 12.0, 3.0 Hz, 1 H, one of CH_2_-1′^a-b^), 5.14 and 5.21 (two m, 1 H, H-2), 7.05–7.69 (multiplets, 12 H, H-2″^a-b^, H-4″^a-b^, H-5″^a-b^, H-7″^a-b^, H-9″^a-b^ and H-10″^a-b^). ^13^C NMR (150 MHz, CDCl_3_:CD_3_OD, 2:1, v/v) δ: 17.22, 17.35, 17.41, 17.46, 17.57 and 17.66 (C-3′^a-b^), 44.42, 44.47, 44.70, 44.79 and 44.83 (C-2′^a-b^), 53.30 and 53.40 (N(CH_3_)_3_), 54.55, 54.57 and 54.61 (O-CH_3_^a-b^), 58.35 (d, *J*_C-P_ = 5.0 Hz, C-α), 58.59 (d, *J*_C-P_ = 4.9 Hz, C-α), 62.13, 62.40 and 62.42 (C-1), 62.96 and 63.03 (two d, *J*_C-P_ = 5.2 Hz, C-3), 63.13 and 63.18 (two d, *J*_C-P_ = 5.6 Hz, C-3), 65.41–65.77 (m, C-β), 70.24–70.47 (m, C-2), 105.04, 105.07 and 105.10 (C-7″^a-b^), 118.34, 118.43 and 118.45 (C-5″^a-b^), 125.27–125.74 (C-2″^a-b^ and C-10″^a-b^), 126.56–128.78 (C-4″^a-b^ and C-9″^a-b^), 128.32, 128.34, 128.43 and 128.45 (C-3″^a-b^), 133.20, 133.28 and 133.30 (C-8″^a-b^), 134.72, 134.87, 134.88, 134.92, 134.95 and 135.00 (C-1″^a-b^), 157.15, 157.21 and 157.23 (C-6″^a-b^), 173.73, 173.81, 173.82, 173.92, 174.09, 174.11, 174.21 and 174.25 (C-1′^a-b^). ^31^P NMR (243 MHz, CDCl_3_:CD_3_OD, 2:1, v/v) δ: –1.01 and –1.19. ESI-MS: *m/z* calculated for C_36_H_44_NO_10_P [M + H]^+^: 682.2781. Found: 682.2760.

### Cells and cell culture

Human promyelocytic leukemia HL-60 cells and human colon cancer cell line Caco-2 were obtained from the American Type Culture Collection (Manassas, VA, USA). The HL-60 cells were cultured in an RPMI medium supplemented with 10% fetal bovine serum (FBS, Cytogen), 2 mM L-glutamine, and antibiotics (complete RPMI). The Caco-2 cells were cultured in an DMED medium supplemented with 20% fetal bovine serum (FBS, Cytogen), 2 mM glutamine and antibiotics (complete DMED). Intestinal epithelial IPEC-J2 cells from newborn porcine, a kind gift from Prof. P. Schierack (Institute of Biotechnology, Faculty of Environment and Natural Sciences, Brandenburg University of Technology Cottbus-Senftenberg, Senftenberg, Germany)^34^ were cultured in DMEM medium supplemented with 10% fetal bovine serum (FBS, Invitrogen, Carlsbad, USA), 2 mM glutamine and antibiotics (complete DMEM).

### WST-1 cell proliferation assay

Cells (4 × 10^3^/well) were grown for 24 h in 96-well plates (Sarstedt, Germany) in appropriate complete medium at 37 °C and atmosphere containing 5% CO_2_. Next day, increased concentrations of analyzed compounds (50, 100, 200 μM) dissolved in dimethyl sulfoxide (DMSO, Chempur, Poland) were added to cells growing in successive wells, and cell cultures were continued for 72 h. Due to the poor solubility of compound 13 in DMSO, its cytotoxicity was not tested. After indicated periods of time, WST-1 mixture was added to each well, and the cells were cultured for additional 6 h. The absorbance at 450 nm was measured on an EnSpire 2300 Multilabel Reader (Perkin-Elmer, USA), and data were analyzed using the program EnSpire 9.0 (Perkin-Elmer, USA). The experiments were performed in triplicates and repeated at least three times independently. Results were presented as mean IC_50_ (concentration of the tested compound, that inhibits cell proliferation by 50%) ± standard deviation. IC_50_ values were calculated using Cheburator 0.4 software^35^.

### Statistical analysis

All statistical analyses were performed using Prism 5.0 (GraphPad, La Jolla, CA, USA). To statistically evaluate the significance of differences between cytotoxicity of reference drugs (ibuprofen and naproxen) and phosphatidylcholines containing ibuprofen or naproxen moieties non-parametric two-way Annova and multicomparsion Bonfferoni post-test was performed. The *p*-value significance threshold was set at 0.01 due to relatively low number of measured replicates. In all analyses, the results were considered statistically significant when *p* < 0.01.

## Electronic supplementary material

Supplementary Information
