# Supplementary material for: Syntheses and cytotoxicity of phosphatidylcholines containing ibuprofen or naproxen moieties
Source: Sci Rep. 2019 Jan 18;9:220. doi: 10.1038/s41598-018-36571-1 (PMC6338774; doi:10.1038/s41598-018-36571-1)
Supplement: Supplementary file 1 — Supplementary Information [file 41598_2018_36571_MOESM1_ESM.pdf]

Supporting Information

Syntheses and cytotoxicity of phosphatidylcholines containing ibuprofen or naproxen moieties

Marek Kłobucki[a], Anna Urbaniak[b], Aleksandra Grudniewska[a], Bartłomiej Kocbach[b], Gabriela Maciejewska[c], Grzegorz Kiełbowicz[a], Maciej Ugorski[b,d], Czesław Wawrzeńczyk[a,*]

[a] Department of Chemistry, Wrocław University of Environmental and Life Sciences, Norwida 25, 50-375 Wrocław, Poland

[b] Laboratory of Glycobiology, Ludwik Hirszfeld Institute of Immunology and Experimental Therapy, Polish Academy of Sciences, Weigla 12, 53-114 Wrocław, Poland

[c] Faculty of Chemistry, Wrocław University of Science and Technology, Wybrzeże Wyspiańskiego 27, 50-370 Wrocław, Poland

[d] Department of Biochemistry and Molecular Biology, Wrocław University of Environmental and Life Sciences, Norwida 31, 50-375 Wrocław, Poland

[*] Corresponding author. Email address: czeslaw.wawrzenczyk@upwr.edu.pl

Content	Page
^{1}H and ^{13}C NMR spectra of **7** (Fig. S1-S2)	2-3
^{1}H and ^{13}C NMR spectra of **8** (Fig. S3-S4)	4-5
^{1}H and ^{13}C NMR spectra of **9** (Fig. S5-S6)	6-7
^{1}H and ^{13}C NMR spectra of **10** (Fig. S7-S8)	8-9
^{1}H and ^{13}C NMR spectra of **12** (Fig. S9-S10)	10-11
^{1}H and ^{13}C NMR spectra of **13** (Fig. S11-S12)	12-13
^{1}H and ^{13}C NMR spectra of **15** (Fig. S13-S14)	14-15
^{1}H and ^{13}C NMR spectra of **16** (Fig. S15-S16)	16-17

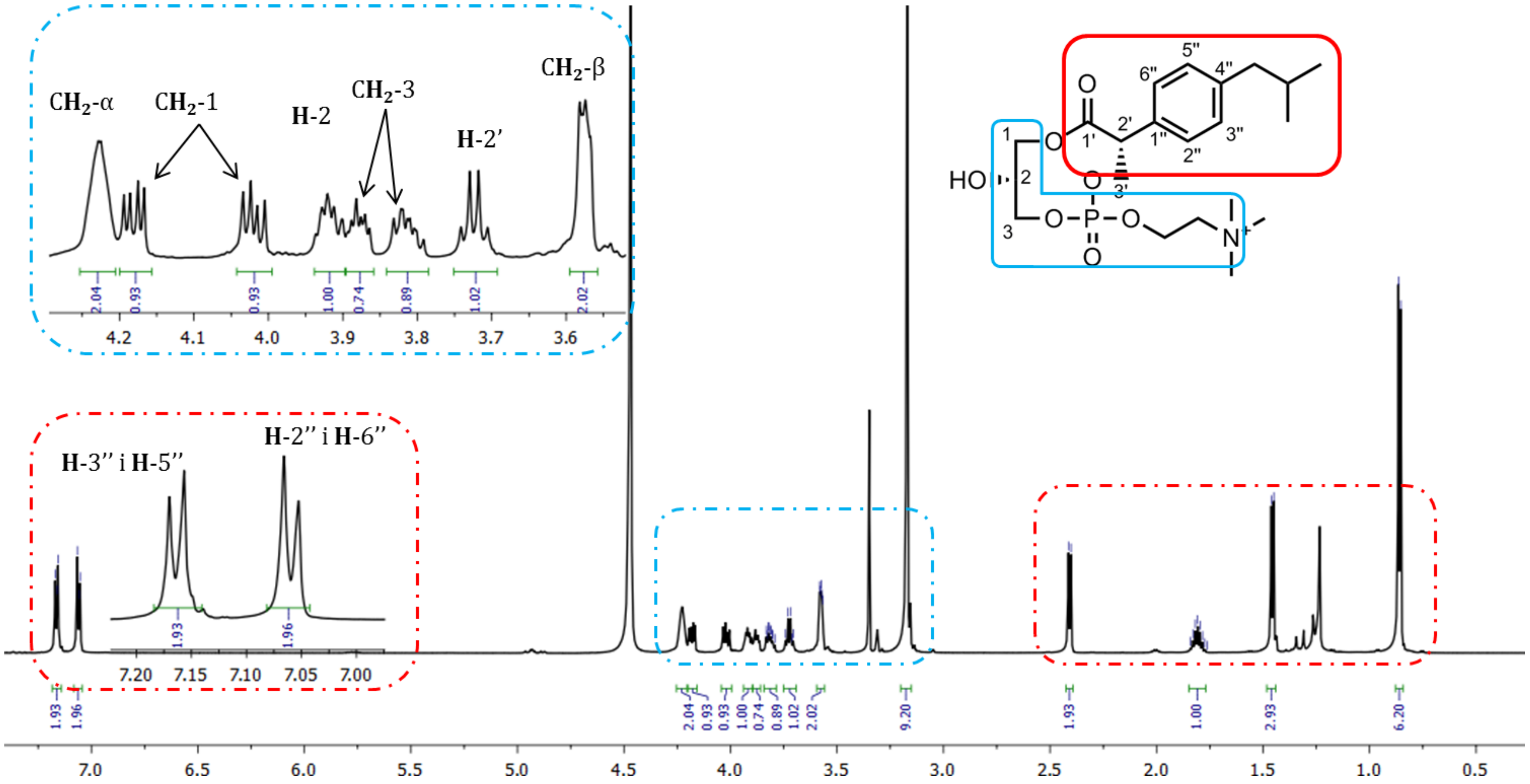

Figure S1. ^{1}H NMR spectrum (600 MHz, $CDCl_3$) of **7**.

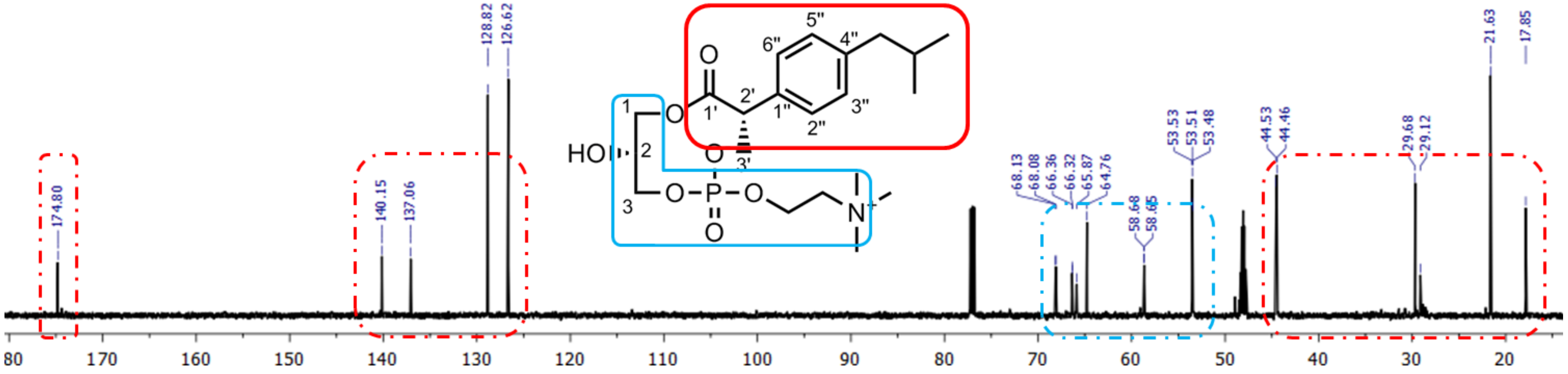

Figure S2. ^{13}C NMR spectrum (150 MHz, CDCl$_3$) of **7**.

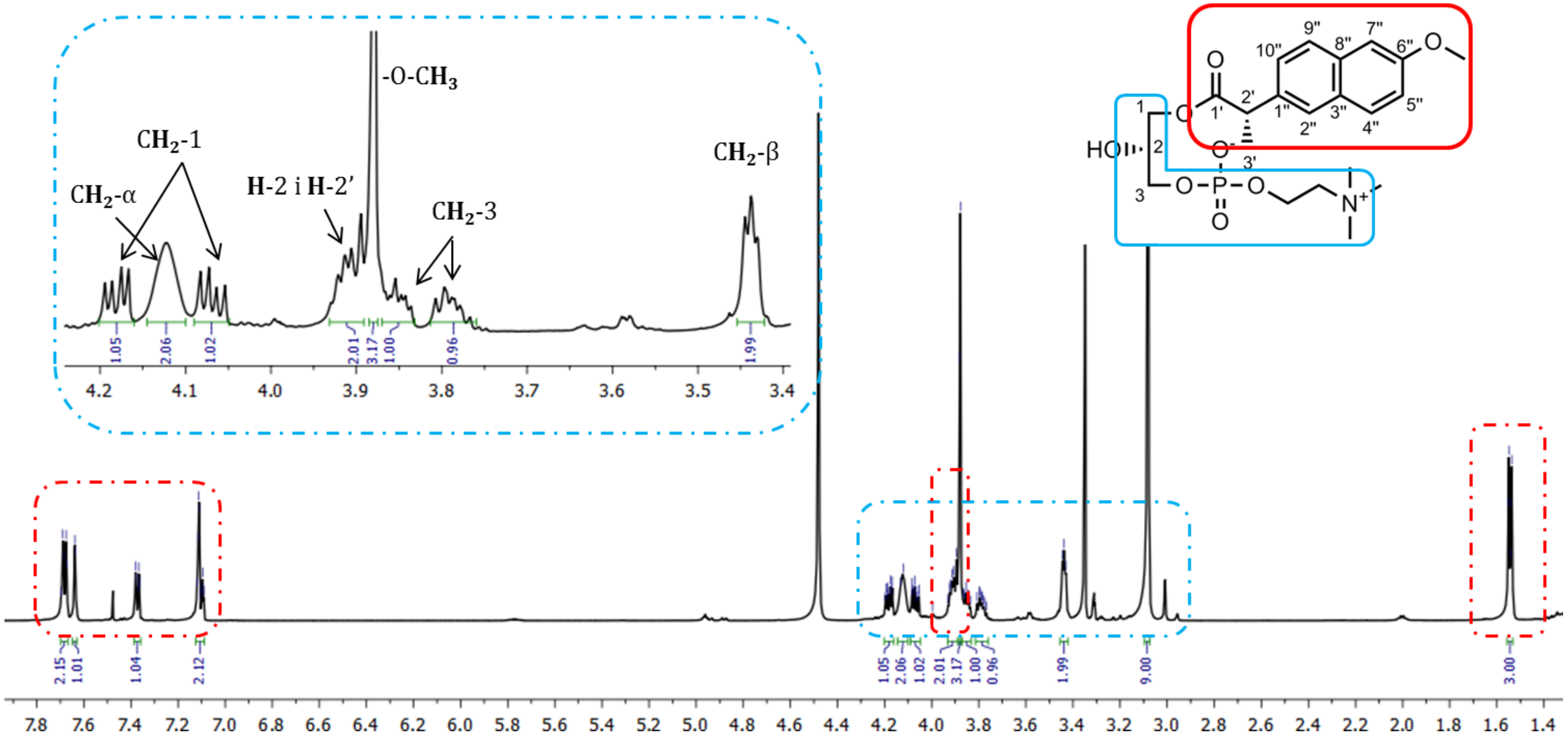

Figure S3. ^{1}H NMR spectrum (600 MHz, $CDCl_3$) of **8**.

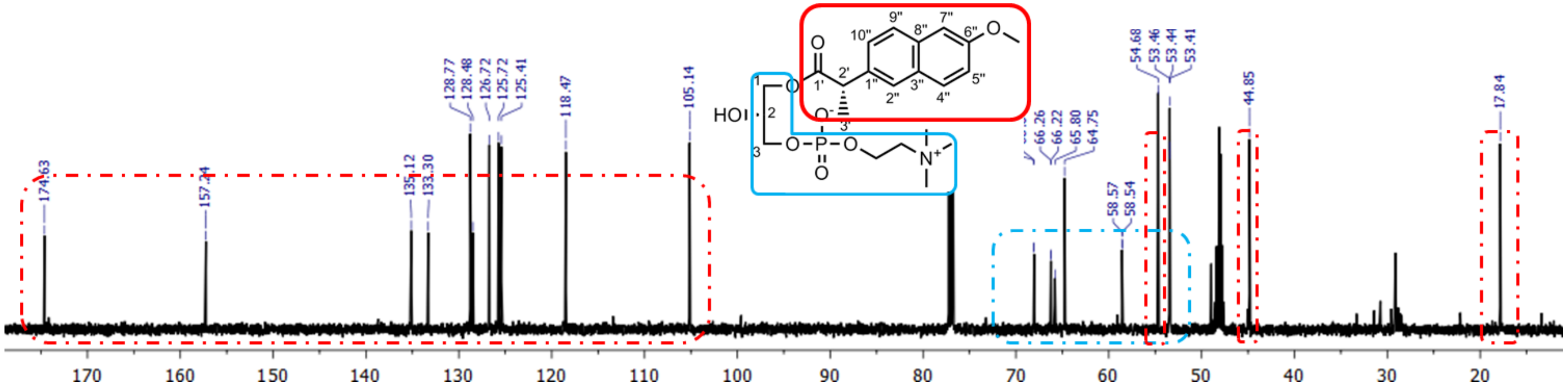

Figure S4. ^{13}C NMR spectrum (600 MHz, $CDCl_3$:CD_3OD, 2:1, v/v) of **8**.

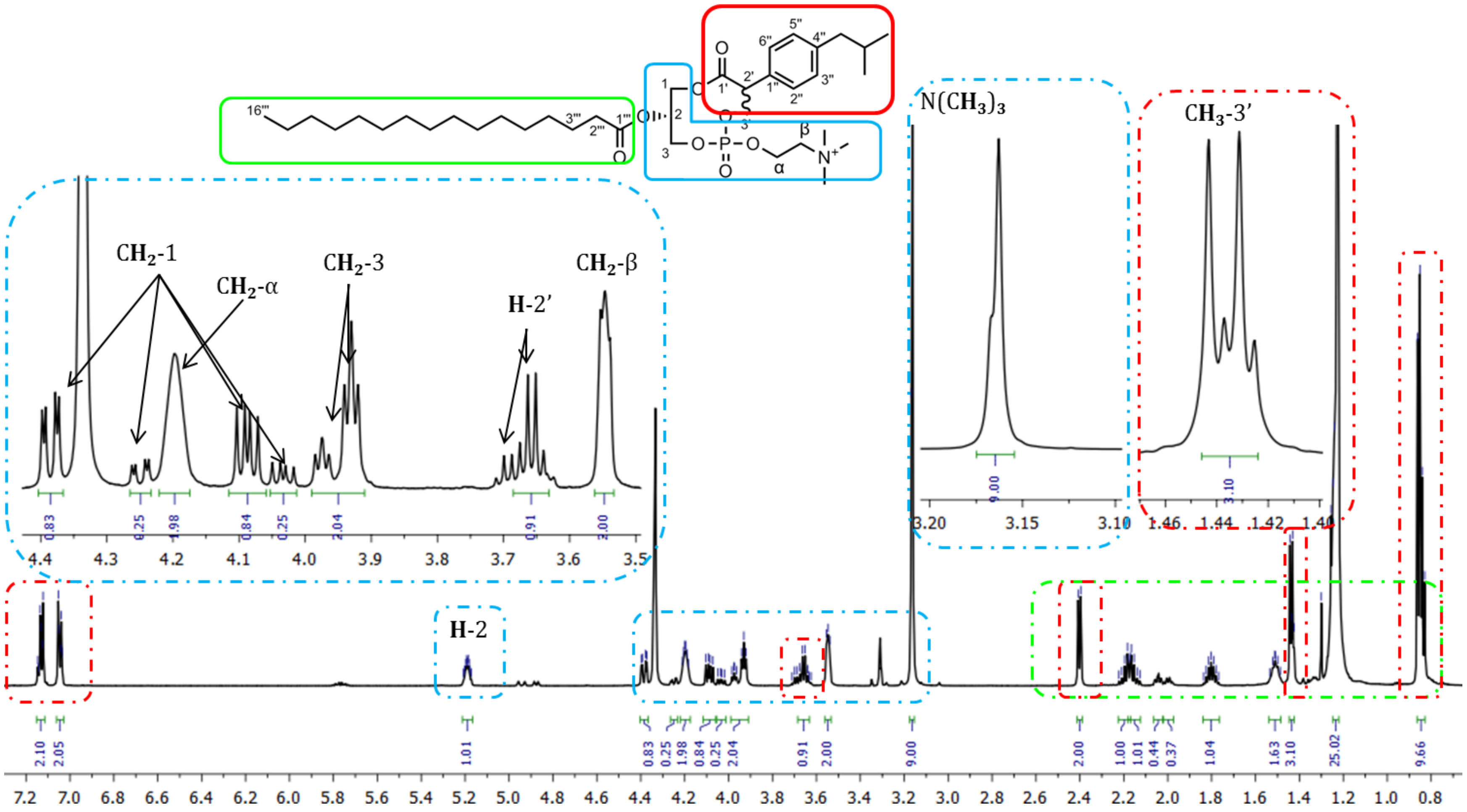

Figure S5. ^{1}H NMR spectrum (600 MHz, $CDCl_3$:CD_3OD, 2:1, v/v) of **9**.

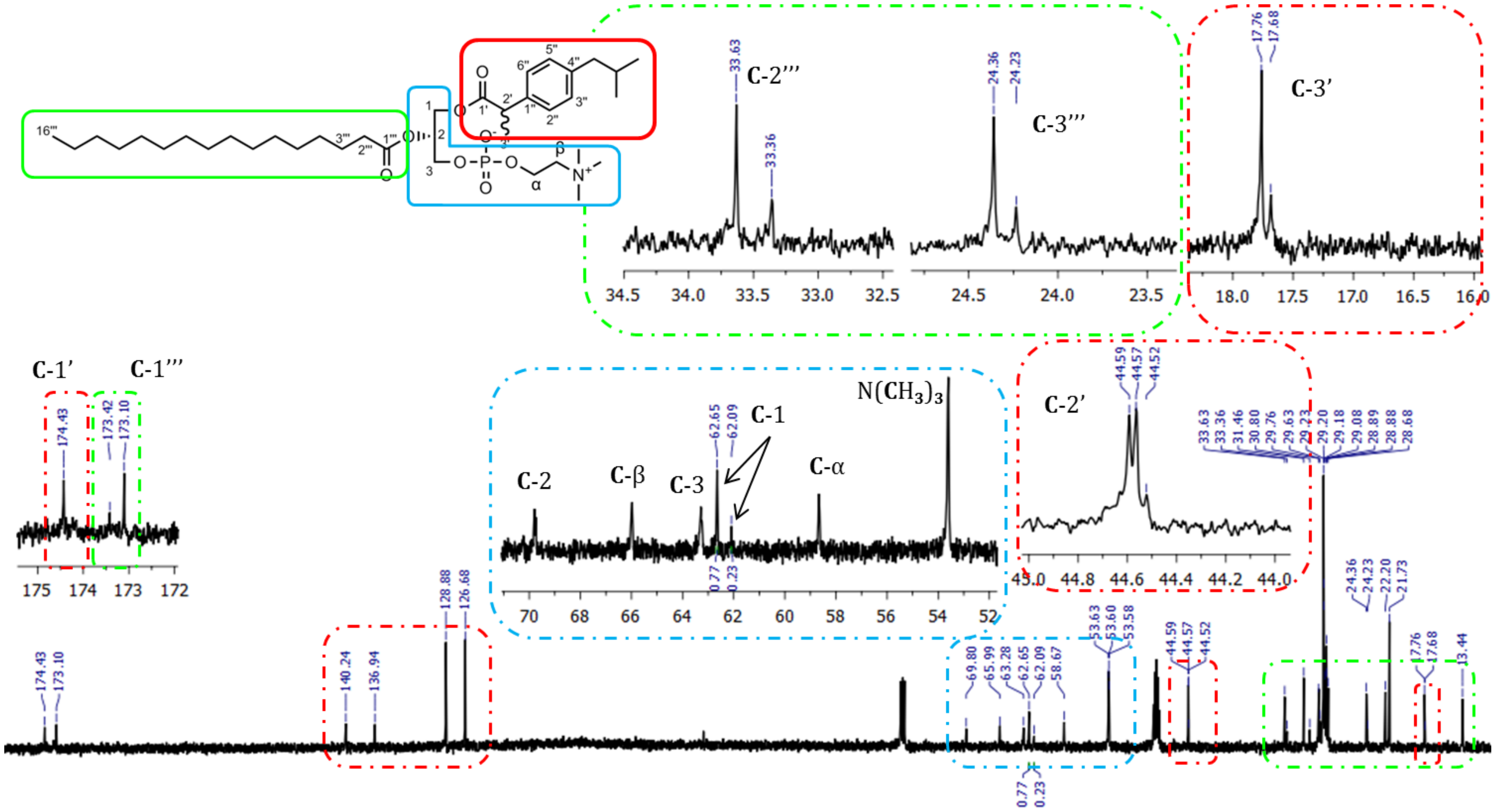

Figure S6. ^{13}C NMR spectrum (150 MHz, $CDCl_3$:CD_3OD, 2:1, v/v) of **9**.

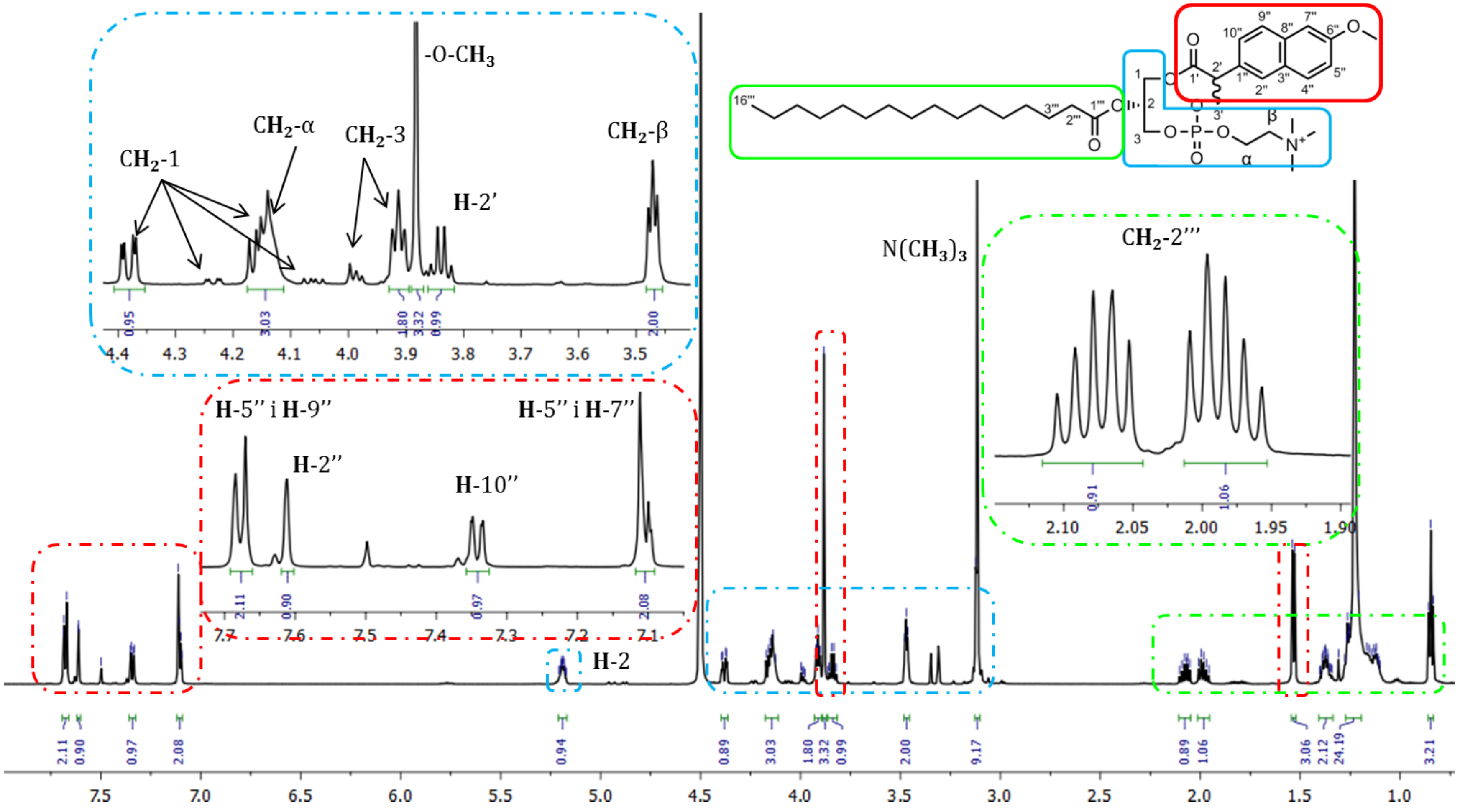

Figure S7. ^{1}H NMR spectrum (600 MHz, $CDCl_3$:CD_3OD, 2:1, v/v) of **10**.

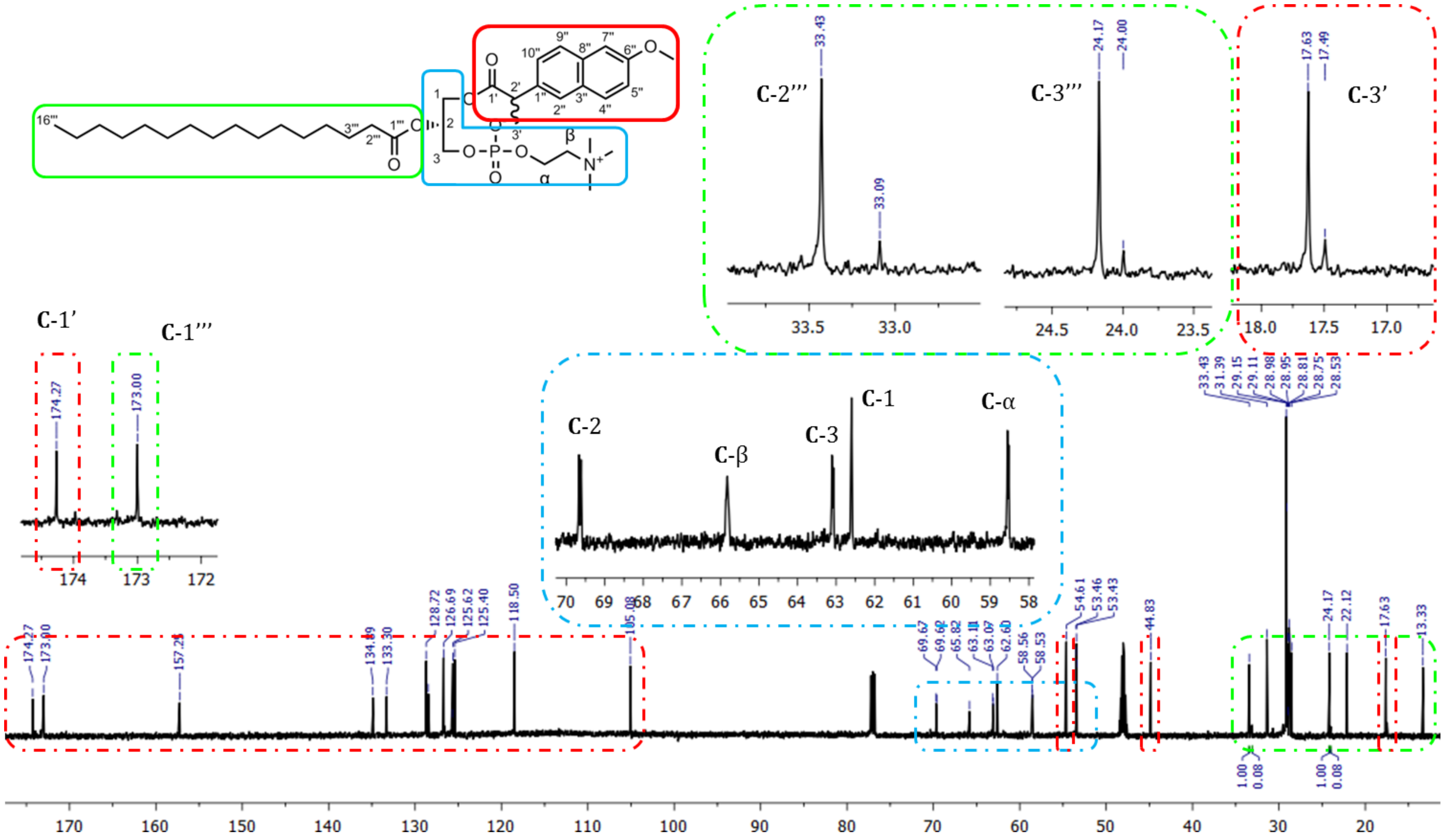

Figure S8. ^{13}C NMR spectrum (150 MHz, $CDCl_3$:CD_3OD, 2:1, v/v) of **10**.

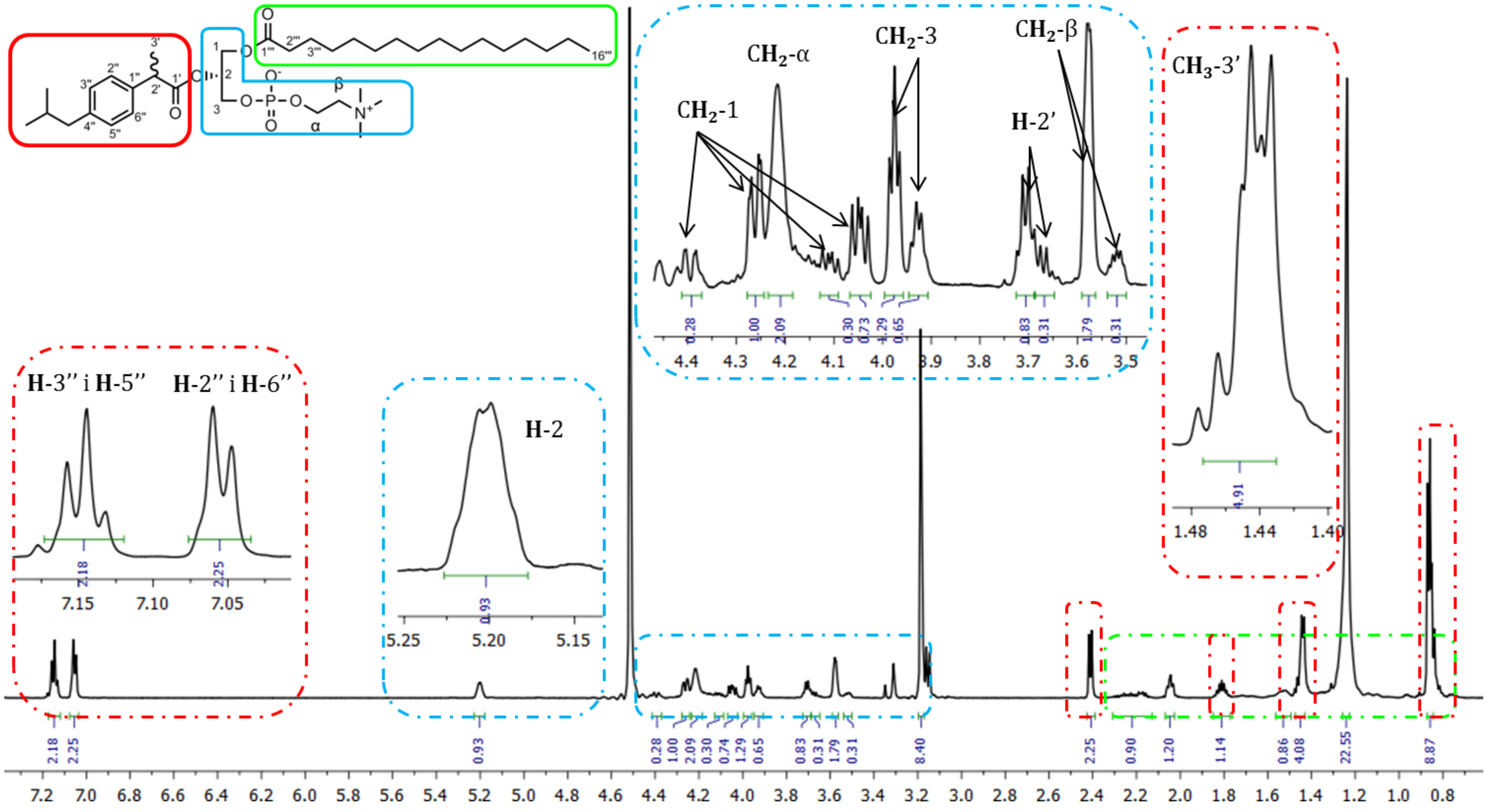

Figure S9. ^{1}H NMR spectrum (600 MHz, $CDCl_3$:CD_3OD, 2:1, v/v) of **12**.

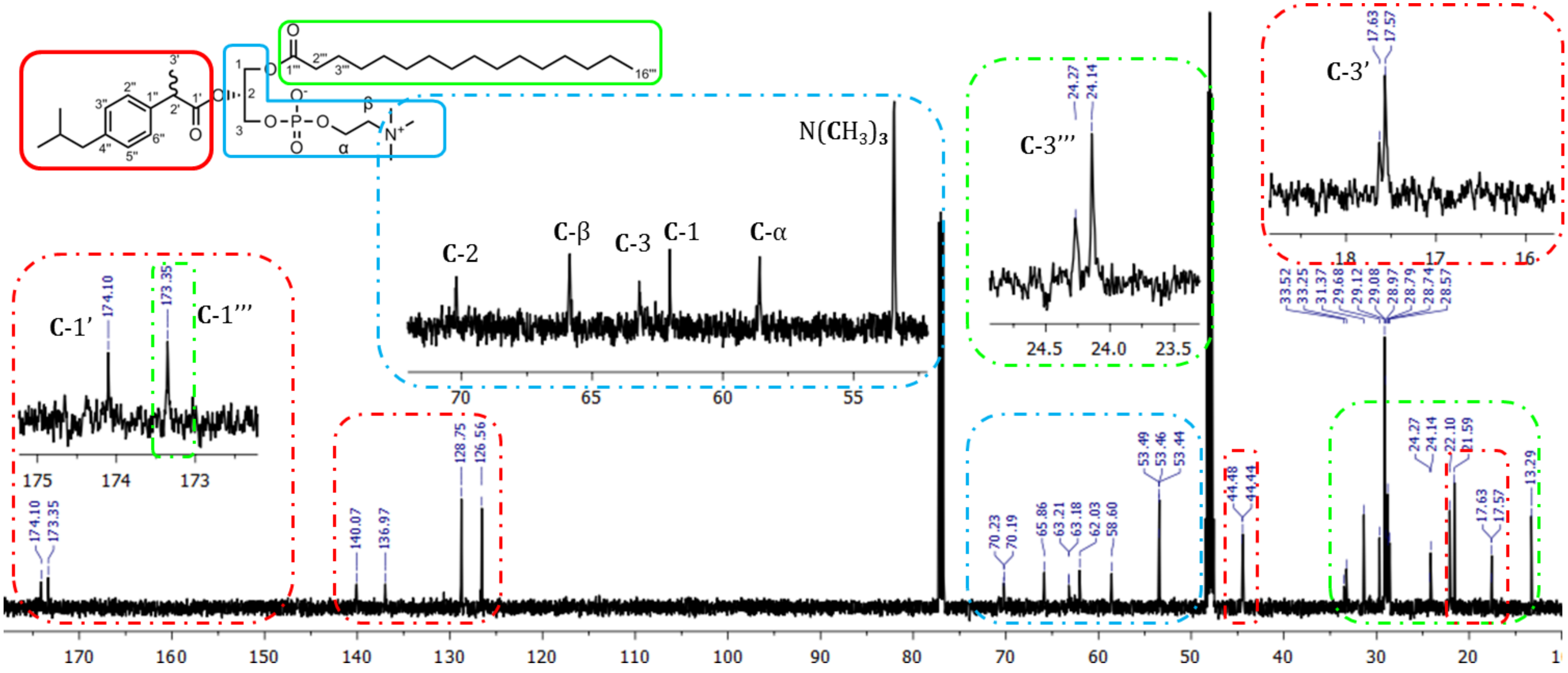

Figure S10. ^{13}C NMR spectrum (150 MHz, $CDCl_3$:CD_3OD, 2:1, v/v) of **12**.

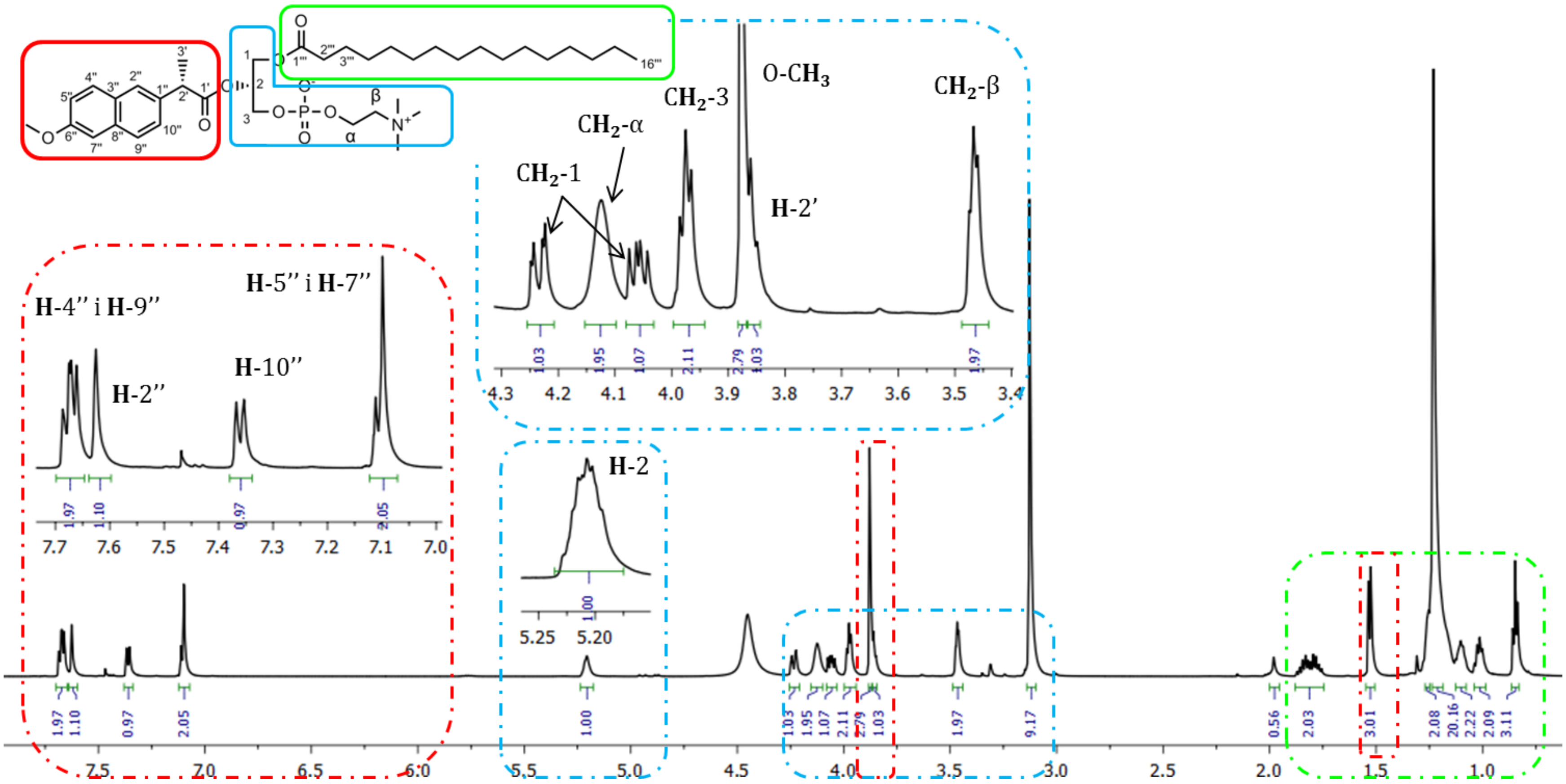

Figure S11. ^{1}H NMR spectrum (600 MHz, $CDCl_3$:CD_3OD, 2:1, v/v) of **13**.

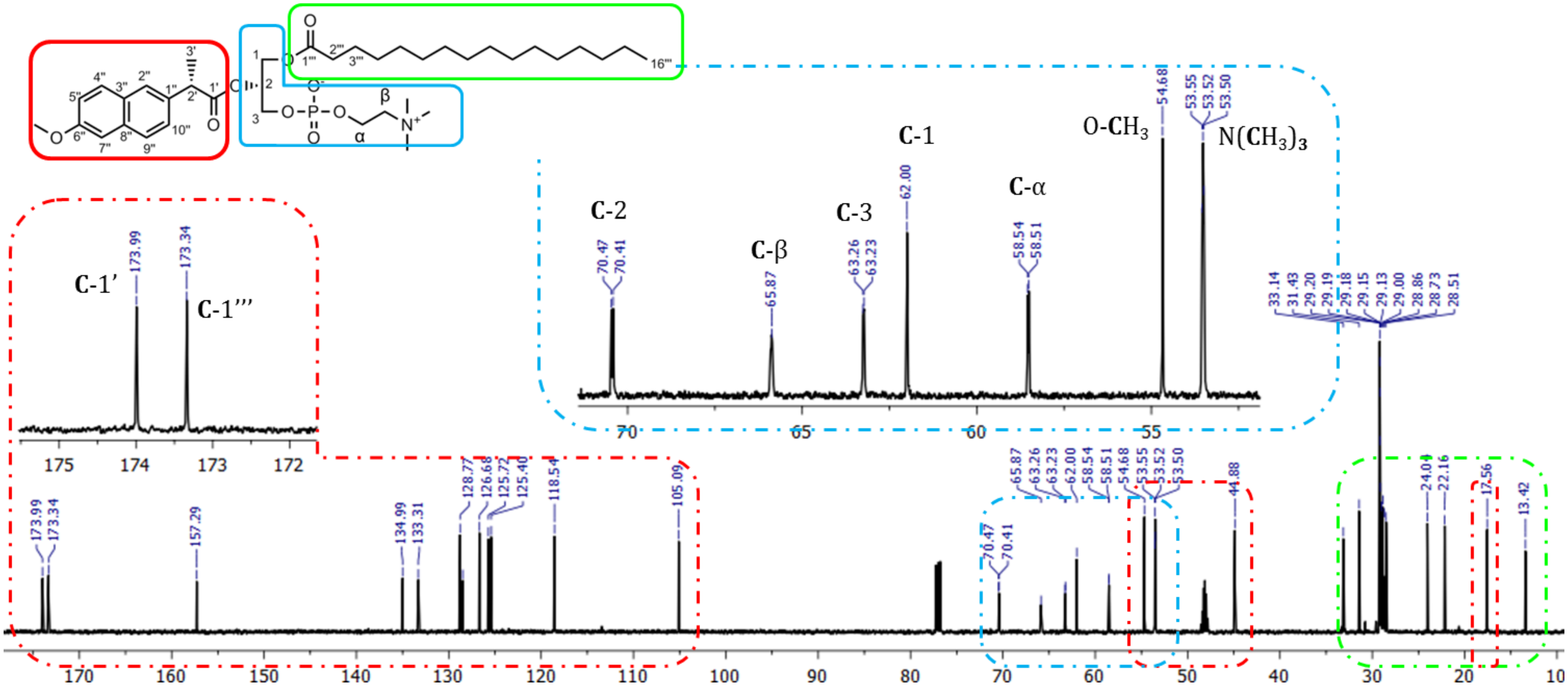

Figure S12. ^{13}C NMR spectrum (150 MHz, $CDCl_3$:CD_3OD, 2:1, v/v) of **13**.

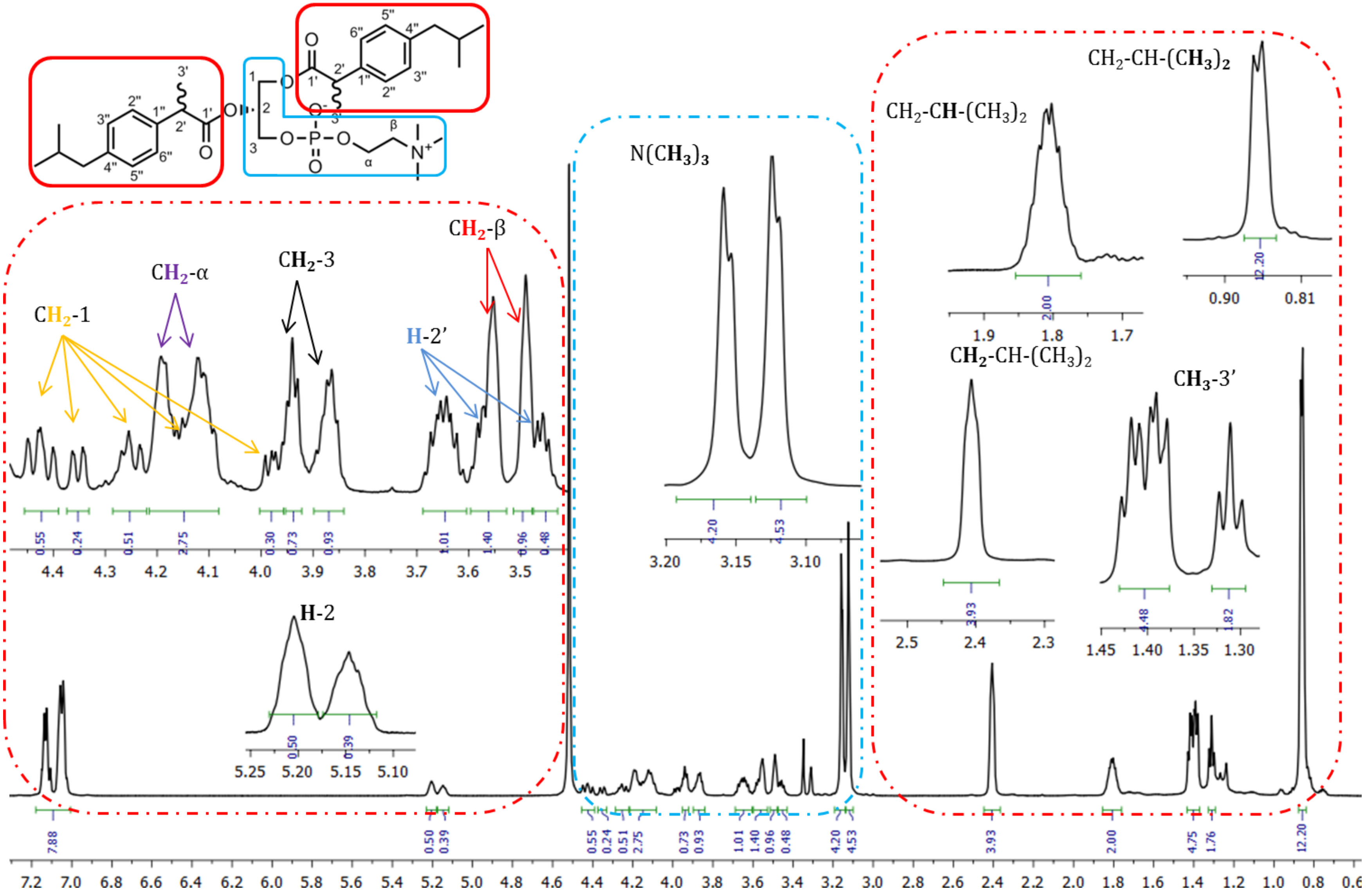

Figure S13. ^{1}H NMR spectrum (600 MHz, $CDCl_3$:CD_3OD, 2:1, v/v) of **15**.

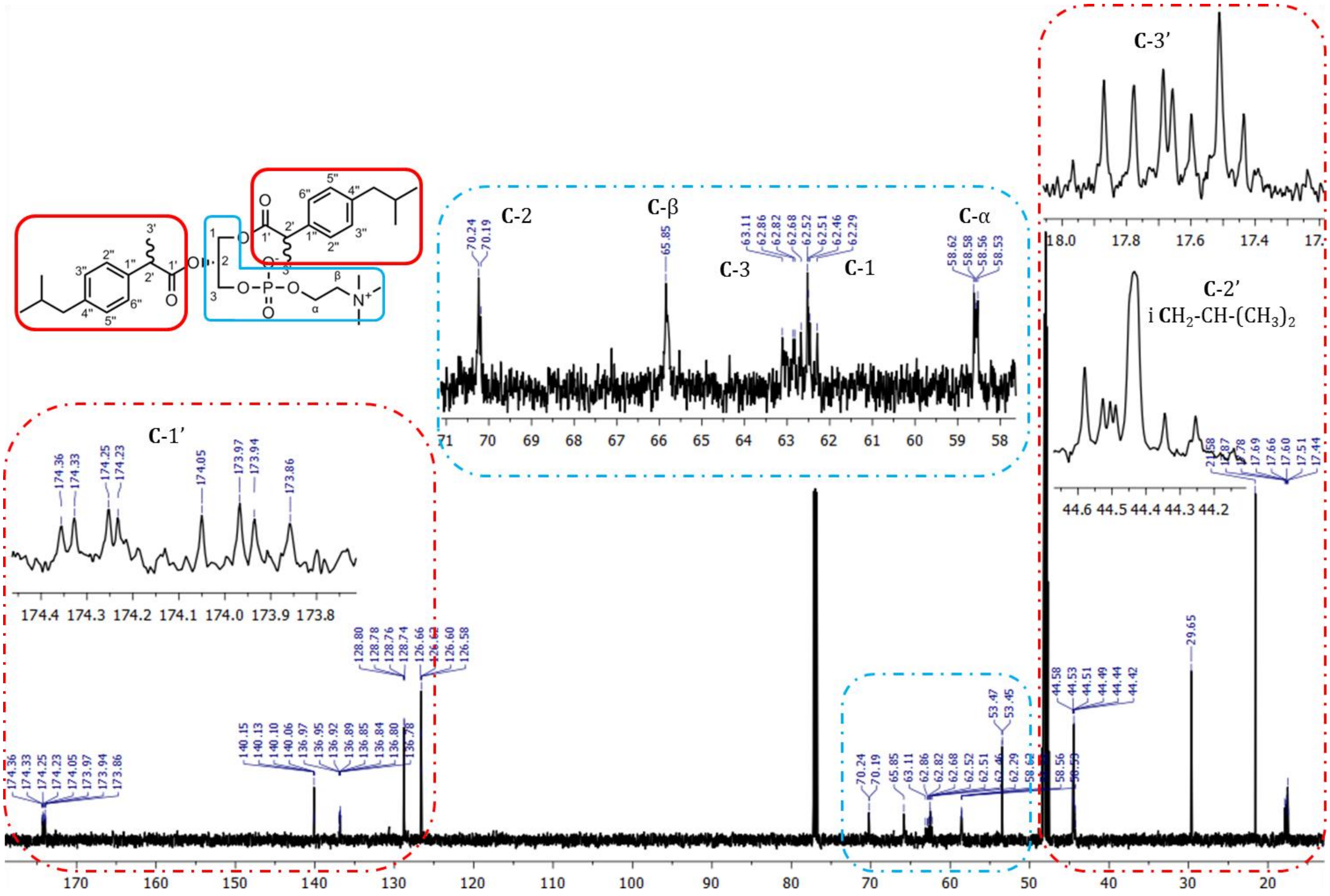

Figure S14. ^{13}C NMR spectrum (150 MHz, $CDCl_3$:CD_3OD, 2:1, v/v) of **15**.

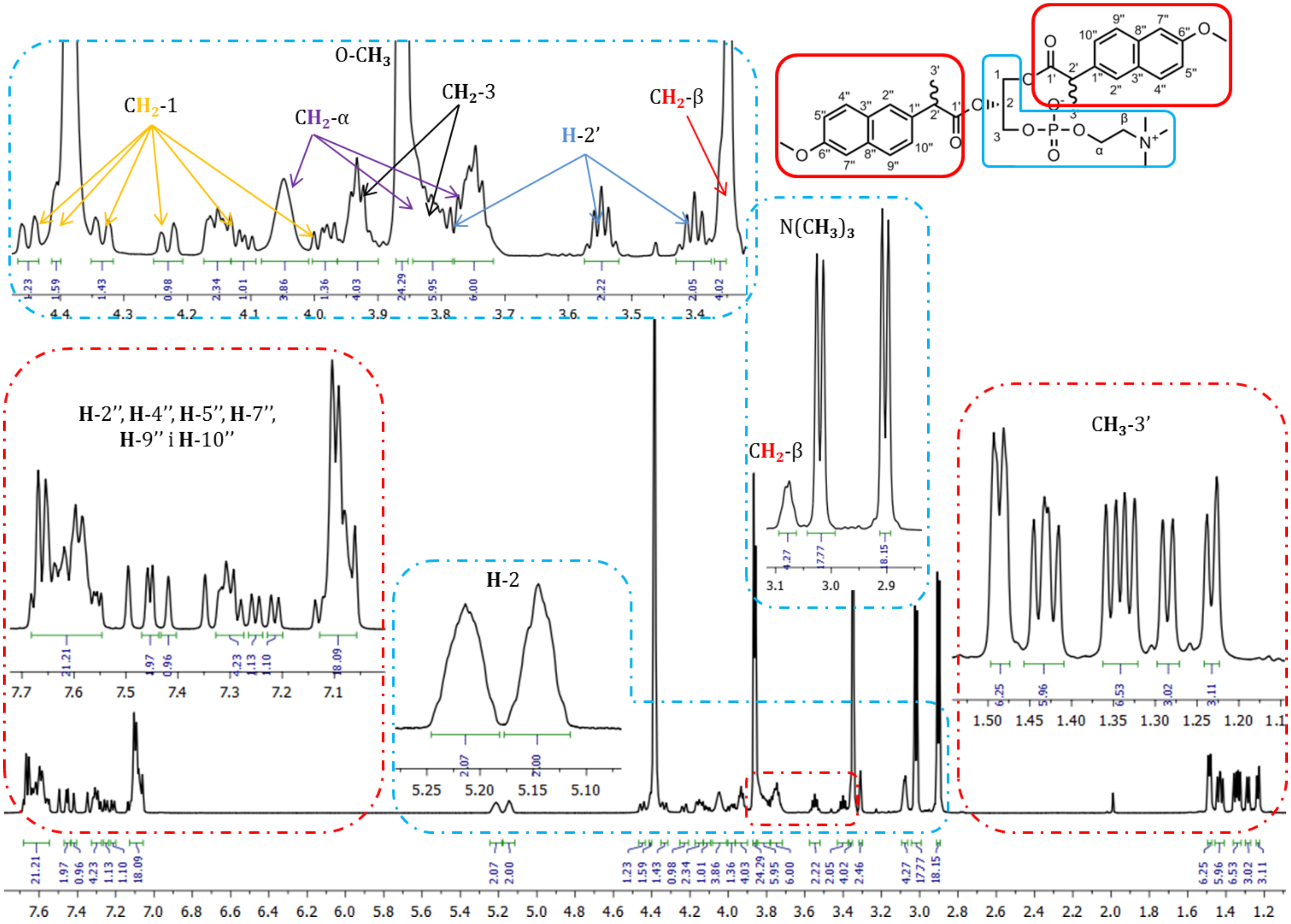

Figure S15. ^{1}H NMR spectrum (600 MHz, $CDCl_3$:CD_3OD, 2:1, v/v) of **16**.

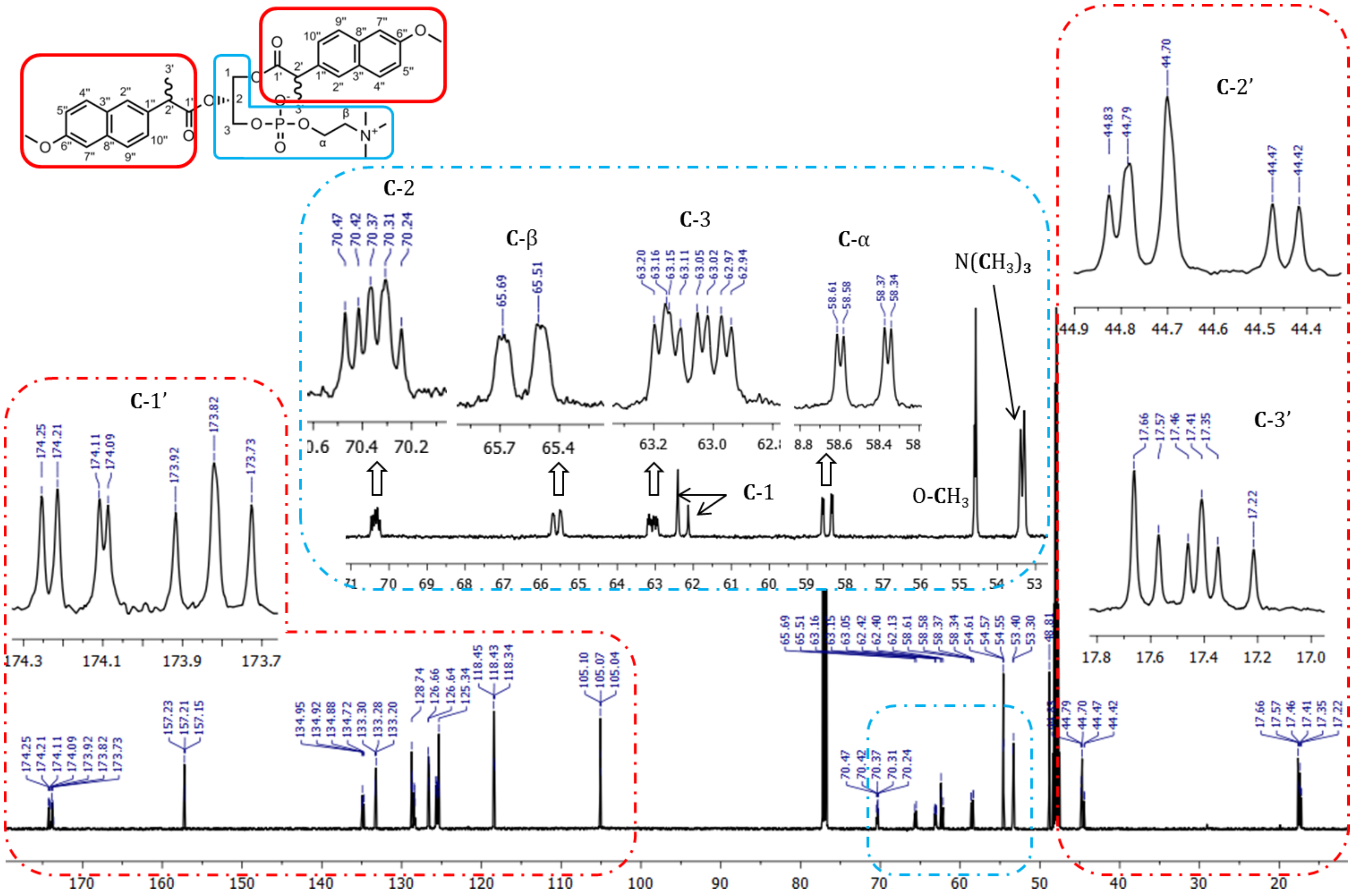

Figure S16. ^{13}C NMR spectrum (150 MHz, $CDCl_3$:CD_3OD, 2:1, v/v) of **16**.
